# Phenotypic and integrated analysis of a comprehensive *Pseudomonas aeruginosa* PAO1 library of mutants lacking cyclic-di-GMP-related genes

**DOI:** 10.3389/fmicb.2022.949597

**Published:** 2022-07-22

**Authors:** Kira Eilers, Joey Kuok Hoong Yam, Richard Morton, Adeline Mei Hui Yong, Jaime Brizuela, Corina Hadjicharalambous, Xianghui Liu, Michael Givskov, Scott A. Rice, Alain Filloux

**Affiliations:** ^1^MRC Centre for Molecular Bacteriology and Infection, Department of Life Sciences, Imperial College London, London, United Kingdom; ^2^Singapore Centre for Environmental Life Sciences Engineering, Nanyang Technological University, Singapore, Singapore; ^3^Department of Medical Microbiology, Amsterdam UMC, Universitair Medische Centra, University of Amsterdam, Amsterdam, Netherlands; ^4^Department of Biology, Institute of Molecular Biology and Biophysics, Eidgenössische Technische Hochschule Zürich, Zurich, Switzerland; ^5^Department of Immunology and Microbiology, Faculty of Health and Medical Sciences, Costerton Biofilm Center, University of Copenhagen, Copenhagen, Denmark; ^6^Commonwealth Scientific and Industrial Research Organisation, Agriculture and Food, Westmead and Microbiomes for One Systems Health, Melbourne, VIC, Australia

**Keywords:** c-di-GMP, *Pseudomonas*, biofilm, diguanylate cyclase, phosphodiesterase

## Abstract

*Pseudomonas aeruginosa* is a Gram-negative bacterium that is able to survive and adapt in a multitude of niches as well as thrive within many different hosts. This versatility lies within its large genome of *ca.* 6 Mbp and a tight control in the expression of thousands of genes. Among the regulatory mechanisms widespread in bacteria, cyclic-di-GMP signaling is one which influences all levels of control. c-di-GMP is made by diguanylate cyclases and degraded by phosphodiesterases, while the intracellular level of this molecule drives phenotypic responses. Signaling involves the modification of enzymes’ or proteins’ function upon c-di-GMP binding, including modifying the activity of regulators which in turn will impact the transcriptome. In *P. aeruginosa*, there are *ca.* 40 genes encoding putative DGCs or PDEs. The combined activity of those enzymes should reflect the overall c-di-GMP concentration, while specific phenotypic outputs could be correlated to a given set of *dgc/pde*. This notion of specificity has been addressed in several studies and different strains of *P. aeruginosa*. Here, we engineered a mutant library for the 41 individual *dgc/pde* genes in *P. aeruginosa* PAO1. In most cases, we observed a significant to slight variation in the global c-di-GMP pool of cells grown planktonically, while several mutants display a phenotypic impact on biofilm including initial attachment and maturation. If this observation of minor changes in c-di-GMP level correlating with significant phenotypic impact appears to be true, it further supports the idea of a local *vs* global c-di-GMP pool. In contrast, there was little to no effect on motility, which differs from previous studies. Our RNA-seq analysis indicated that all PAO1 *dgc/pde* genes were expressed in both planktonic and biofilm growth conditions and our work suggests that c-di-GMP networks need to be reconstructed for each strain separately and cannot be extrapolated from one to another.

## Introduction

Bacteria have evolved complex strategies to adapt to their environment. *Pseudomonas aeruginosa* is an ubiquitous Gram-negative pathogen thriving in the environment (soil/water) and infecting hosts, e.g., humans, animals, plants, and insects (Crone et al., 2020). It causes acute as well as chronic infections that can last for the host’s lifetime (e.g., in the cystic fibrosis lung; Lund-Palau et al., 2016; Moradali et al., 2017; Smith et al., 2017). Eight per cent of the *P. aeruginosa* genome encodes regulatory genes, which result in expression or silencing of specific molecular determinants and adaptation to changing environmental conditions (Rodrigue et al., 2000).

In recent years the “extraordinaire” intracellular messenger c-di-GMP has been shown to operate as a central switch that controls bacterial behaviors (Romling et al., 2013; Dahlstrom and O’Toole, 2017; Jenal et al., 2017). The paradigm is that at high c-di-GMP concentrations, a biofilm lifestyle is favored, while lowering the concentration triggers motility and virulence. However, the paradox is that a single molecule controls a multitude of outputs (e.g., motility, virulence, biofilm, antimicrobial resistance), while at the same time dozens of enzymes making and breaking c-di-GMP (diguanylate cyclases, DGCs (Ryjenkov et al., 2005) and phosphodiesterases, PDEs (Schmidt et al., 2005) respectively) are encoded in the *P. aeruginosa* genome (Valentini et al., 2018). Given the high number of PDEs and DGCs present, it has been hypothesized that the flow of c-di-GMP might go through a variety of transmission paths, each delivering a unique phenotypic outcome. For example, it was reported that c-di-GMP may bind an effector/receptor (Ryjenkov et al., 2006; Lory et al., 2009), protein or riboswitch (Reyes-Darias and Krell, 2017), changing its conformation and/or activity to generate an output. Indeed, c-di-GMP signaling was discovered through its binding and triggering of cellulose synthase activity (Ross et al., 1985). In addition to binding to a specific protein or RNA, c-di-GMP has been suggested to control a variety of phenotypes in the same organism by using a range of different approaches (Boyd and O’Toole, 2012). Among these are (i) differential gene (*dgc*/*pde*) expression; (ii) different affinity between c-di-GMP and receptors/effectors; (iii) local instead of global intracellular pools of c-di-GMP (Hengge, 2021); and (iv) direct transmission of c-di-GMP from the DGC onto a receptor/effector or, conversely, direct removal from the receptor/effector by the PDE.

c-di-GMP-mediated gene expression and the related phenotypes in a bacterium appear to be quite complex, making predictions about what phenotypes might be controlled by a specific DGC or PDE difficult. To further complicate matters, some species have a limited number of DGCs/PDEs while others have an abundance. Even within a species, *de novo* prediction of the c-di-GMP regulatory network based on related strains also appears to be challenging. In *P. aeruginosa*, there are *ca.* 40 DGCs/PDEs encoded on the genome of any single strain (Kulasakara et al., 2006), with some variation. For example, PAO1 encodes the PDE Arr (Hoffman et al., 2005) while PA14 does not; and PA14 encodes the PDE PvrR while PAO1 does not (Drenkard and Ausubel, 2002). This strain-level difference in c-di-GMP-controlling proteins is also apparent in clinical strains, suggesting it is common for *P. aeruginosa*. For example, there are clinical isolates that show some specific c-di-GMP features such as the cystic fibrosis isolate CF39S that carries the thermosensory diguanylate cyclase TdcA, which is absent in both PAO1 and PA14 (Almblad et al., 2021). Thus, prediction of the c-di-GMP network for one strain based on other strains may not be accurate. Furthermore, direct comparison between PAO1 and PA14 would not be possible since PA14 has a mutation in *ladS*, a gene encoding a sensor involved in the Gac/Rsm network (Mikkelsen et al., 2011), which was shown to be instrumental for the *P. aeruginosa* lifestyle switch (Ventre et al., 2006) in direct connection with the c-di-GMP network (Moscoso et al., 2011). Even when looking at one single strain of *P. aeruginosa*, e.g., PAO1, it was shown that significant differences and genetic drift can be observed when comparing strains issued from one laboratory or another (Chandler et al., 2019), which may subsequently result in significant differences, including in c-di-GMP signaling.

Thus to obtain a fully integrated vision of c-di-GMP signaling in *P. aeruginosa* there is still a need to test the individual *dgc* or *pde* mutants. Therefore, this study has engineered a library of 41 individual *dgc* or *pde* mutants from our PAO1 laboratory strain. This library was used to assess the most common c-di-GMP-related phenotypes, biofilm formation and motility (Coggan and Wolfgang, 2012). The most impacted phenotype in this strain of PAO1 was bacterial attachment and biofilm maturation, while motility was generally not affected, in contrast with the current literature. Moreover, we showed that all *dgc/pde* genes were expressed in PAO1 in both planktonic and biofilm growth conditions. While the deletion mutants showed distinct phenotypic changes, the intracellular concentration of c-di-GMP was minor. This suggests that, in many cases, a change in the local pool of c-di-GMP is more likely to drive a phenotypic change than large variations in the global pool.

## Materials and methods

### Bacterial strains and growth conditions

Bacterial strains and plasmids used in this study are described in Supplementary Tables S1, S2, respectively. *P. aeruginosa* strains were grown in tryptone soy broth (TSB; Sigma), lysogeny broth (LB) or ABTG (15.1 mM (NH_4_)_2_SO_4_; 33.7 mM Na_2_HPO_4_.2H_2_O; 22 mM KH_2_PO_4_; 50 mM NaCl; 1 mM MgCl_2_.6H_2_O; 100 μM CaCl_2_.2H_2_O; 1 μM FeCl_3_.6H_2_O; 0.4 g of glucose per liter) supplemented with antibiotics where appropriate (Gentamicin 100 μg/ml or Streptomycin 2,000 μg/ml) at 37°C with agitation. *Escherichia coli* strains were grown in LB supplemented with antibiotics where appropriate (Kanamycin 50 μg/ml, Streptomycin 50 μg/ml or Gentamicin 50 μg/ml).

### DNA manipulation

DNA purification was performed using the PureLink Genomic DNA minikit (Life Technologies), while plasmid DNA isolation was performed using the QIAprep spin miniprep kit (Qiagen). Restriction endonucleases were used according to the manufacturer’s specifications (New England Biolabs or Roche) and all used oligonucleotides are listed in Supplementary Table S3 and were purchased from Sigma. KOD Hot Start DNA Polymerase (Novagen) was used to amplify genes or DNA fragments used for the construction of mutator plasmids and deletion mutants as described by the manufacturer, with the inclusion of 1 M betaine (Sigma). Colony PCR was performed with Taq polymerase (New England Biolabs). DNA sequencing was performed by GATC Biotech.

### Construction of *Pseudomonas aeruginosa* mutants

*P. aeruginosa* deletion mutants were constructed as described previously (Vasseur et al., 2005) using the suicide plasmid pKNG101 (Kaniga et al., 1991). Note that the method was slightly modified and used Vogel-Bonner Minimal (VBM) medium instead of PIA. Briefly, to create a clean deletion in the PAO1 strain, 500 bp DNA fragments of the 5′ (upstream) and 3′ (downstream) ends of the gene of interest were obtained by PCR using PAO1 chromosomal DNA as a template. The upstream fragment was amplified with the oligonucleotides P1 and P2 while the downstream fragment was amplified using P3 and P4 (Supplementary Table S3). A third PCR step using P1 and P4 resulted in a DNA fragment with a clean deletion of the gene. The gene fragments were cloned into pCR-BluntII-TOPO (Invitrogen), their sequences were confirmed and sub-cloned into the pKNG101 suicide vector (Supplementary Table S2). The pKNG-derivatives were maintained in *E. coli* strain CC118λpir and mobilized into *P. aeruginosa* PAO1 using *E. coli* 1047 carrying the conjugative plasmid pRK2013 (Figurski and Helinski, 1979). Clones in which double recombination events occurred, resulting in the deletion of the gene of interest, were isolated using counterselection on sucrose plates as previously described (Vasseur et al., 2005). Gene deletions were verified by PCR using external primers P5 and P6 (Supplementary Table S3).

### Crystal violet attachment assay

The attachment assay was adapted from previously published methods (Merritt et al., 2005). In brief, overnight cultures were adjusted to an OD_600_ of 0.2, 10 μl were subsequently inoculated into 96-well plates containing 190 μl LB and incubated at 37°C without shaking for 6 h. Wells were washed three times with distilled water and attached cell material was then stained with 0.1% w/v crystal violet solution (5% methanol, 5% isopropanol in ddH2O). After staining, wells were washed three times with distilled water to remove excess dye and crystal violet was dissolved in 20% acetic acid solution. Absorbance of dissolved crystal violet was measured at 595 nm. Assays were performed with eight wells/strain and in three biological replicates.

### Flow cell biofilm

The flow cell system was adapted from previously published methods (Sternberg and Tolker-Nielsen, 2006). GFP-tagged *P. aeruginosa* biofilms were cultivated in 40 mm × 4 mm × 1 mm three-channel flow cells with ABTG medium at 37°C. Briefly, the overnight cultures were centrifuged at 13,000 g for 3 min to remove supernatant, pellets were resuspended in ABTG medium and adjusted to OD_600_ ~ 0.05 (inoculum). Using syringe and needle, 500 μl of inoculum was injected into each flow cell channel (3 channels per strain). The flow cells were placed in an inverted position for 1 h incubation before being reverted to an upright position, and were supplied with ABTG medium at the flow rate of 4 ml/h using a Cole-Parmer peristaltic pump (Cole Parmer Instrument Co., Germany). After 72 h of cultivation, the ABTG medium supply was stopped.

The biofilms were observed using a LSM780 confocal laser scanning microscope (CLSM; Carl Zeiss, Germany). Seven microscopy images per channel were captured and acquired using 20x/0.80 DICII objective lens and a 488 nm argon multiline laser was used to monitor the GFP-expressing bacterial cells. The excitation and emission wavelengths for GFP are 488 nm and 509 nm, respectively. The acquired images were further processed using IMARIS version 9 (Bitplane AG, Zurich, Switzerland). Experiments were performed in triplicate and representative images are shown. The acquired microscopy images were processed using COMSTAT2 software^1^ to measure the three-dimensional biofilm image stack (Heydorn et al., 2000). The three parameters used in COMSTAT2 software to analyze the biofilm structures were biomass, mean thickness and roughness coefficient. All COMSTAT2 parameters were fixed at default settings prior to image analysis. Experiments were performed as biological triplicates and results are shown as the mean ± s.d.

### Motility assays

Motility assays were carried out as previously described (Rashid and Kornberg, 2000; Mikkelsen et al., 2009). Swimming assays were conducted on 10 g/l tryptone, 5 g/l NaCl, 0.2% agar (Oxoid) plates. 500 nl of standardized overnight culture (grown in LB medium) were injected below the surface into the middle of the agar and plates were incubated at 37°C overnight (16 h) before the swimming diameter was measured. Swimming assays were performed with 5 technical and 3 biological triplicates.

Twitching assays were performed on 1% LB agar plates and bacteria were inoculated by picking a colony using a sterile tip and stabbing to the bottom of the plates, which were then incubated at 37°C for 48 h. The agar was subsequently peeled off and cells were stained with crystal violet and the twitch diameter was measured. Twitching assays were performed in 3 technical and 3 biological triplicates.

### Intracellular c-di-GMP measurement

For c-di-GMP quantification, samples were prepared as described previously (Spangler et al., 2010) and analyzed by liquid-chromatography mass spectrometry (LC–MS/MS). In brief, *P. aeruginosa* strains were grown for 6 h in 10 ml of LB medium to stationary phase and cells were harvested by centrifugation. Collected cells were resuspended in 200 μl extraction solution (Acetonitrile/methanol/water, 2/2/1, v/v/v), incubated on ice for 15 min and heated for 10 min at 95–99°C. Cells were centrifuged for 10 min at 4°C, 20,800 g, and supernatant fluid was collected. Extraction was repeated twice and supernatants from the three extraction steps were combined and incubated at −20°C overnight. Extraction fluids were centrifuged again, and supernatant fluid was analyzed at the BIOLOG Life Science Institute (Biolog, Bremen) *via* LC–MS/MS. Samples were compared to a standard curve derived from measurements of known concentrations of pure c-di-GMP to determine the concentration (in nM) of c-di-GMP in the samples, and the data were normalized to the total protein content of the sample determined by Bradford assay. For each strain, experiments were done in biological duplicates and LC–MS/MS measurements were repeated in duplicate. Data are presented as pmol c-di-GMP/mg of total protein.

### RNA-seq analysis

*Pseudomonas aeruginosa* cells were either grown in planktonic condition in ABTG medium for 5 h at 37°C or under biofilm settings in flow tubes with ABTG for 72 h. The total RNA from *P. aeruginosa* cells was extracted using Qiagen RNeasy Mini Kit (catalog number: 74104, Qiagen^®^, Germany) according to the manufacturer’s instructions. Extracted RNA samples were treated with DNase using Turbo DNA-free^™^ kit (catalog number: AM1907, Thermo Fisher Scientific, United States) and purified DNA-free RNA samples were subjected to ribosomal depletion using the Ribo-Zero^™^ Magnetic Kits (Illumina, United States). RNA and DNA were quantified using the Qubit^™^ RNA Assay Kits and Qubit^™^ dsDNA HS Assay Kits (Invitrogen, United States), respectively and integrity of RNA was analyzed *via* gel electrophoresis. Complementary DNAs (cDNAs) were reverse transcribed using a NEBNext RNA first and second strand synthesis module (NEB^®^, United States). cDNAs were subjected to Illumina’s TruSeq Stranded mRNA protocol. The quantitated libraries were then pooled at equimolar concentrations and sequenced on an Illumina HiSeq2500 sequencer in rapid mode at a read-length of 100 bp paired-ends. RNA samples were prepared in triplicate from three independent biological samples.

The quality of reads was first assessed using FastQC v0.11.7 (Leggett et al., 2013). Ribosomal RNA reads were discarded using SortMeRNA v4.2.0 (Kopylova et al., 2012) and the remaining non-rRNA reads were trimmed using Trimmomatic v0.38 (Bolger et al., 2014) and mapped against the *P. aeruginosa* PAO1 reference genomes (GenBank accession numbers: AE004091.2) using HISAT2 v2.2.1 (Kim et al., 2019). Aligned data were sorted with SAMTools v1.13 (Li et al., 2009) and used as input for the *feature counts* function from the Rsubread v2.4.3 package (Liao et al., 2019) to generate a matrix of annotated genes with their corresponding raw counts (Liao et al., 2014, 2019). An average of 95.46% reads were successfully mapped to the reference genome. The count data were then analyzed in both planktonic and biofilm conditions to look for differential gene expression levels and statistical significance using DESeq2 v1.30.1 (Love et al., 2014). Genes with absolute value of Log2 fold change (Log2FC) ≥1 and adjusted value of *p* ≤0.05 were considered as DEGs in comparative analysis. For a system level interpretation, clusterProfiler v3.18.1 (Yu et al., 2012) was used for GO and KEGG pathway enrichment. GO annotation was downloaded from The *Pseudomonas* Genome Database (Winsor et al., 2016).^2^ RNA-seq data are available from the National Center for Biotechnology Information/GEO repository under accession no. GSE203348.

## Results

### Construction of the PAO1 mutant library for genes encoding diguanylate cyclases and phosphodiesterases

Cellular c-di-GMP levels in *P. aeruginosa* and many other bacteria are driven by enzymes that carry any of three essential modules (Ryan et al., 2009; Wei et al., 2019). c-di-GMP makers, DGCs, usually contain a GGDEF domain, whereas c-di-GMP breakers, PDEs, harbor an EAL or HD-GYP domain. In addition, there is a class of proteins that contain both a GGDEF and an EAL domain, giving them the possibility of having DGC or PDE activity, both or neither. In the latter case, GGDEF and EAL domains are often degenerated and may be used to bind c-di-GMP (Navarro et al., 2009; Newell et al., 2009).

**Figure 1:**
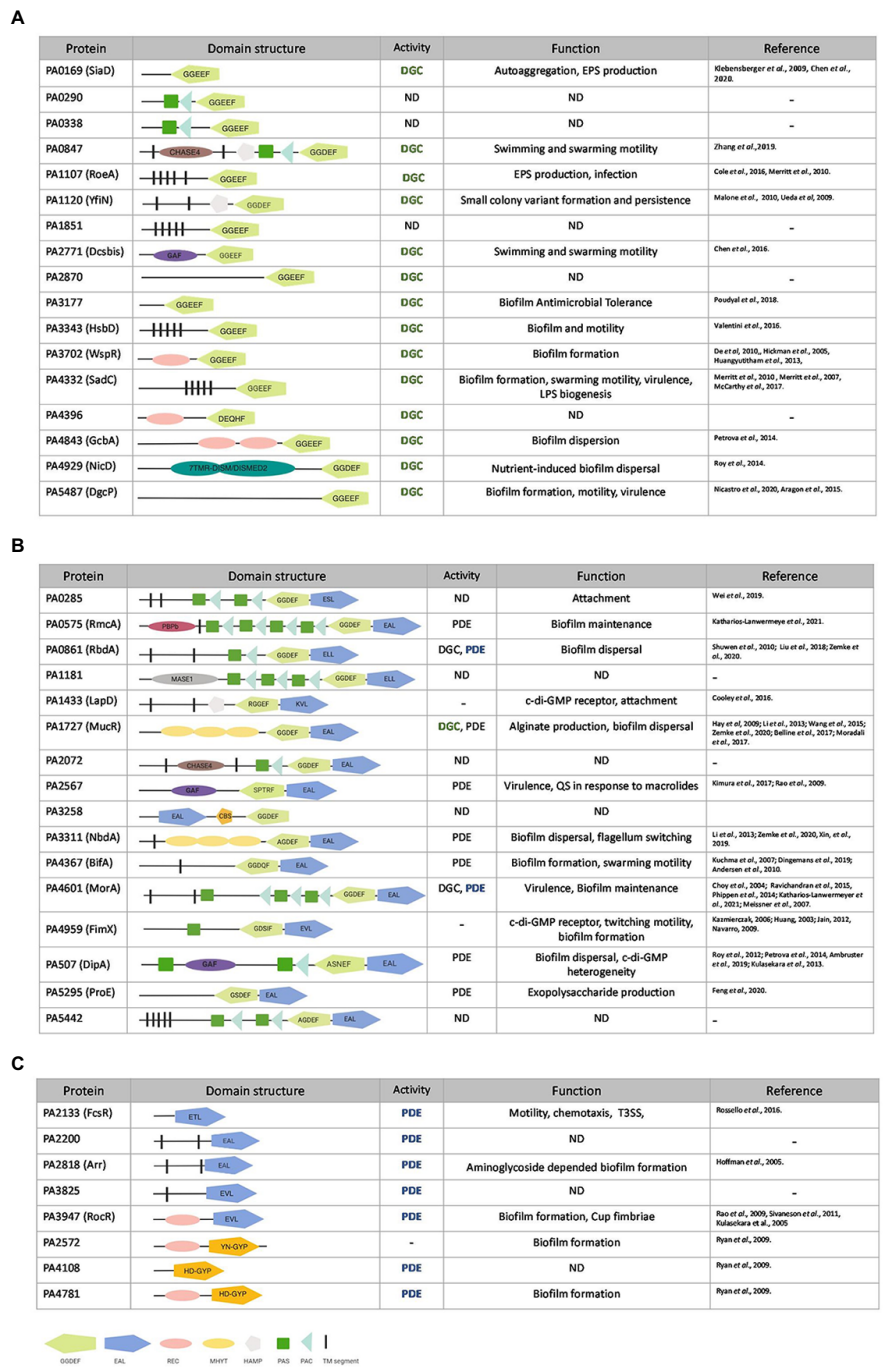
*Pseudomonas aeruginosa* genes encoding putative diguanylate cyclases (DGCs) or phosphodiesterases (PDEs). Genes encoding for GGDEF **(A)**, GGDEF/EAL dual domain **(B)** or EAL/HD-GYP **(C)** motifs in *P. aeruginosa* PAO1. GGDEF domains are shown in light green, EAL domain in dark blue, and HD-GYP in orange. Accessory domains are indicated in pink (REC domain), yellow (MYHT domain), silver (HAMP domain), gray (MASE1), purple (GAF), brown (CHASE4), dark green boxes (PAS domain), light blue (PAC domain), magenta (PBPb domain), and black segments (transmembrane domain). PDE (phosphodiesterase), DGC (diguanylate cyclase) or not yet determined (ND) activity as well as experimentally assessed function (bold and colored) are described and corresponding studies listed.

The annotated genome of *P. aeruginosa* PAO1 (Stover et al., 2000) contains 41 genes that encode 17 proteins with a GGDEF domain, 8 with an EAL or HD-GYP domain and 16 that carry both (Kulasakara et al., 2006; Figure 1). Here two questions arise: why are there so many enzymes with a similar function, and how can a coordinated and specific output associated with any one of these enzymes be generated? The current concept is that the opposing activities of DGCs and PDEs maintain a basal level of c-di-GMP (Wei et al., 2019), but it can be fine-tuned by selective activation or inhibition of a subset in response to specific extracellular cues (Galperin et al., 2001). The domain structure of these c-di-GMP metabolizing enzymes, as determined by the NCBI conserved domain (CD) search tool (RPS-BLAST),^3^ revealed that many of the enzymes are fused to one or more signal-sensing domains at their N-terminus. Out of the 41 GGDEF/EAL/HD-GYP domain-containing proteins, 12 harbor a PAS domain (Per-ARNT-Sim; Delgado-Nixon et al., 2000; Ho et al., 2000) including several that have multiple PAS domains, 6 contain a REC domain (CheY-homologous receiver domain; Huangyutitham et al., 2013) including GcbA that has two of them, 3 carry a GAF domain and 3 a HAMP domain (present in Histidine kinases, Adenyl cyclases, Methyl-accepting proteins and Phosphatases; Aravind and Ponting, 1999; Figure 1). The presence of these regulatory domains suggests that individual DGCs and PDEs exert precise control over c-di-GMP levels in response to various environmental cues.

To provide an integrated and systematic characterization of the contribution of the 41 individual putative DGCs and PDEs to the essential lifestyle transition of *P. aeruginosa* from its motile, planktonic cell state to sessile biofilm communities, we engineered a mutant library in our laboratory PAO1 strain, which has been referred to as PAO1 “Lausanne” since originating from the Dieter Haas laboratory (Heurlier et al., 2004). Each individual *dgc* or *pde* gene was deleted in this background as described in materials and methods. The clean deletions are less likely to generate polar effects as could be the case with transposon libraries. This complete single deletion library of all 41 c-di-GMP metabolizing genes in *P. aeruginosa* PAO1 was subsequently subjected to the methodical assessment of biofilm and motility phenotypes.

### A large majority of c-di-GMP-related genes have an impact on initial attachment to surfaces

Controlling the state of multicellularity in *P. aeruginosa* is a hallmark of c-di-GMP signaling (Valentini and Filloux, 2016). The transition from motile single cellular state to surface-committed multicellular community is initiated by attachment to abiotic surfaces, which is achieved through mechano-sensing *via* the flagellum (Laventie et al., 2019), type IV pili (T4P; O’Toole and Kolter, 1998) and other extracellular appendices such as Cup fimbriae (Vallet et al., 2001; Ruer et al., 2007). This timeframe of initial attachment is crucial for later mature formation of biofilm structures.

**Figure 2:**
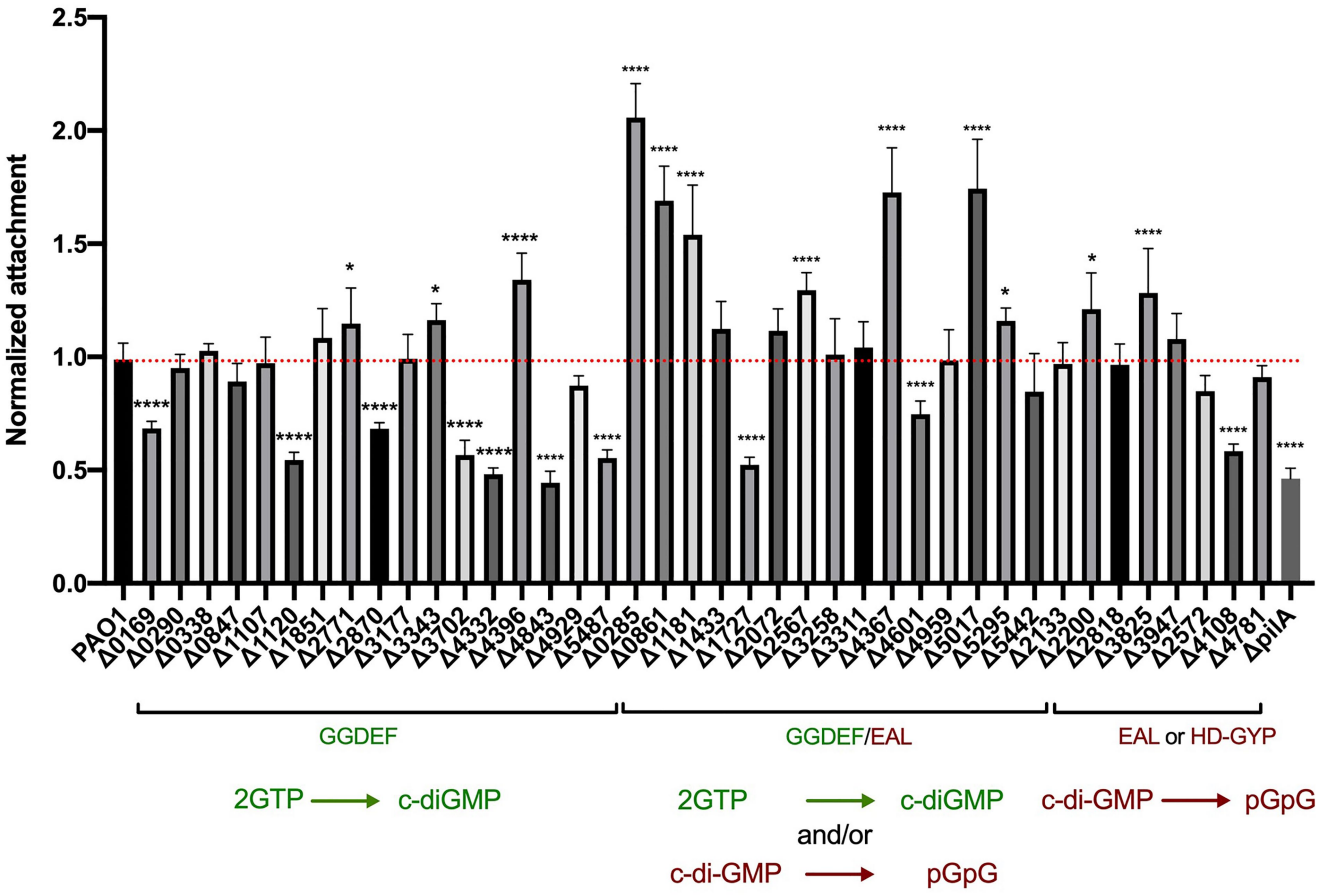
Deletion of genes encoding c-di-GMP metabolizing proteins results in altered attachment profiles. Attachment properties of *Pseudomonas aeruginosa* PAO1 WT and deletion mutants of the 41 genes encoding for GGDEF domains, GGDEF/EAL dual domains as well as EAL or HD-GYP domains. Cells were grown statically at 37°C in LB in 96 wells plates for 6 h, liquid culture was removed and attached cells were stained with crystal violet. Absorbance of crystal violet was measured and is indicative of attached cells. Attachment was assessed in biological triplicates and normalized to PAO1 WT attachment. (Student’s *t*-test, ^*^p < 0.05; and ^****^p < 0.0001).

To quantify the attachment during early biofilm formation, all 41 mutants were tested in a crystal violet assay. Each strain was grown overnight in LB at 37°C and diluted to an OD_600_ of 0.01 in 200 μl in a 96-well polystyrene plate. Plates were incubated under static growth conditions for 6 h at 37°C and planktonic cells removed. Adherent cell mass was washed and stained with crystal violet with the absorbance measured at 595 nm (OD_595_) for quantification of the biomass (Figure 2). Each deletion mutant was assessed in eight technical and three biological replicates. Of the 41 c-di-GMP mutants tested, 22 showed significantly altered attachment behavior. Deletion of genes encoding proteins containing GGDEF domains mainly showed a decrease in attachment, which correlates with previous findings that DGC increases the level of c-di-GMP, which subsequently triggers biofilm formation. As such, mutation of an active DGC should decrease the c-di-GMP level and in turn reduce attachment. This was clearly observed for 7 mutations, PA0169, PA1120 (*yfiN*; Malone et al., 2010), PA2870, PA3702 (*wspR*; Huangyutitham et al., 2013), PA4332 (*sadC*; Merritt et al., 2007, 2010; Moscoso et al., 2014; McCarthy et al., 2017), PA4843 (*gcbA*; Petrova et al., 2014) and PA5487 (*dgcP*; Aragon et al., 2015; Nicastro et al., 2020), among which several have been shown to be active DGCs (Figure 1). Three of the mutants in a putative *dgc* gene resulted in a slight upregulation of attachment (fold change ranging between 1.2 and 1.4): PA2771 (*dcsbis*; Chen et al., 2016), PA3343 (*hsbD*; Valentini et al., 2016) and most significantly PA4396. This could suggest that some DGCs might transiently elevate the level of c-di-GMP, which in turn activates determinants involved in biofilm inhibition or dispersal to build up a potential negative feedback loop. It should be noted that previous studies have shown that PA2771 has very little impact on biofilm (Chen et al., 2016), which would agree with the observation that our mutant did not display any reduction in attachment.

Deletion of genes encoding proteins with GGDEF/EAL tandem domains mostly led to an increase in bacterial attachment (Figure 2). This reflects the fact that most of the dual domain-harboring proteins have been shown to be PDEs and thus the deletion of the corresponding genes would result in an increase of c-di-GMP and consequently an increase in early biofilm attachment. Out of the 16 mutants, six had a significantly elevated attachment (*p* < 0,0001): PA0285, PA0861 (*rbdA*; An et al., 2010; Liu et al., 2018; Zemke et al., 2020), PA1181, PA2567 (Rao et al., 2009; Kimura et al., 2017), PA4367 (*bifA*; Kuchma et al., 2007; Dingemans et al., 2019; Andersen et al., 2021) and PA5017 (*dipA*; Roy et al., 2012; Kulasekara et al., 2013). Interestingly, PA1727 (*mucR*; Hay et al., 2009) and PA4601 (*morA*; Choy et al., 2004; Meissner et al., 2007; Phippen et al., 2014; Ravichandran et al., 2015) exhibited a decrease in attachment (Figure 2), which suggests that not all dual enzymes have a PDE activity, but that some are DGCs. It should be noted that the conditions of the assay may impact on the main activity of the enzyme and that some of the dual enzymes may have both activities, operating as a PDE or a DGC under different conditions (Feirer et al., 2015). Both MorA/PA4601 (Phippen et al., 2014) and MucR/PA1727 (Hay et al., 2009) were shown to have both activities (Figure 1), but in our assay the DGC activity seems to be dominant. RbdA/PA0861 (An et al., 2010) was also shown to have both activities (Figure 1); however, in our assay, it displays a dominant PDE activity. Finally, some dual domain-containing proteins appeared to have neither activity but instead can bind c-di-GMP, e.g., PA1433/LapD (Newell et al., 2009; Cooley et al., 2016) and PA4959/FimX (Huang et al., 2003; Kazmierczak et al., 2006; Navarro et al., 2009). In our assay neither mutant displayed any change in attachment to the abiotic surface.

The last category of c-di-GMP metabolizing proteins contain either an EAL motif or an HD-GYP domain and are proposed to have PDE activity. Out of the 8 proteins in this category, deletion of two genes encoding EAL-harboring enzymes, PA2200 and PA3825, resulted in elevated attachment levels, as would be expected for genes encoding PDEs, although the impact was less than was seen for genes encoding dual domain-containing proteins such as PA0285. A mutant lacking the previously characterized PDE RocR (PA3947; Kulasekara et al., 2005; Rao et al., 2009; Sivaneson et al., 2011) did not show any clear phenotypic outcome in the conditions of the assay. Finally, deletion of PA4108, encoding an HD-GYP-containing protein (Stelitano et al., 2013), resulted in lower attachment, which does not agree with previous work suggesting that HD-GYP-carrying enzymes have PDE activity. This again may reflect specific conditions in our assay and the indirect impact of c-di-GMP levels on downstream events that may be part of negative feedback loops (Xu et al., 2019).

### Deletion of c-di-GMP metabolism-related genes results in heterogenous biofilm architecture profiles

**Figure 3:**
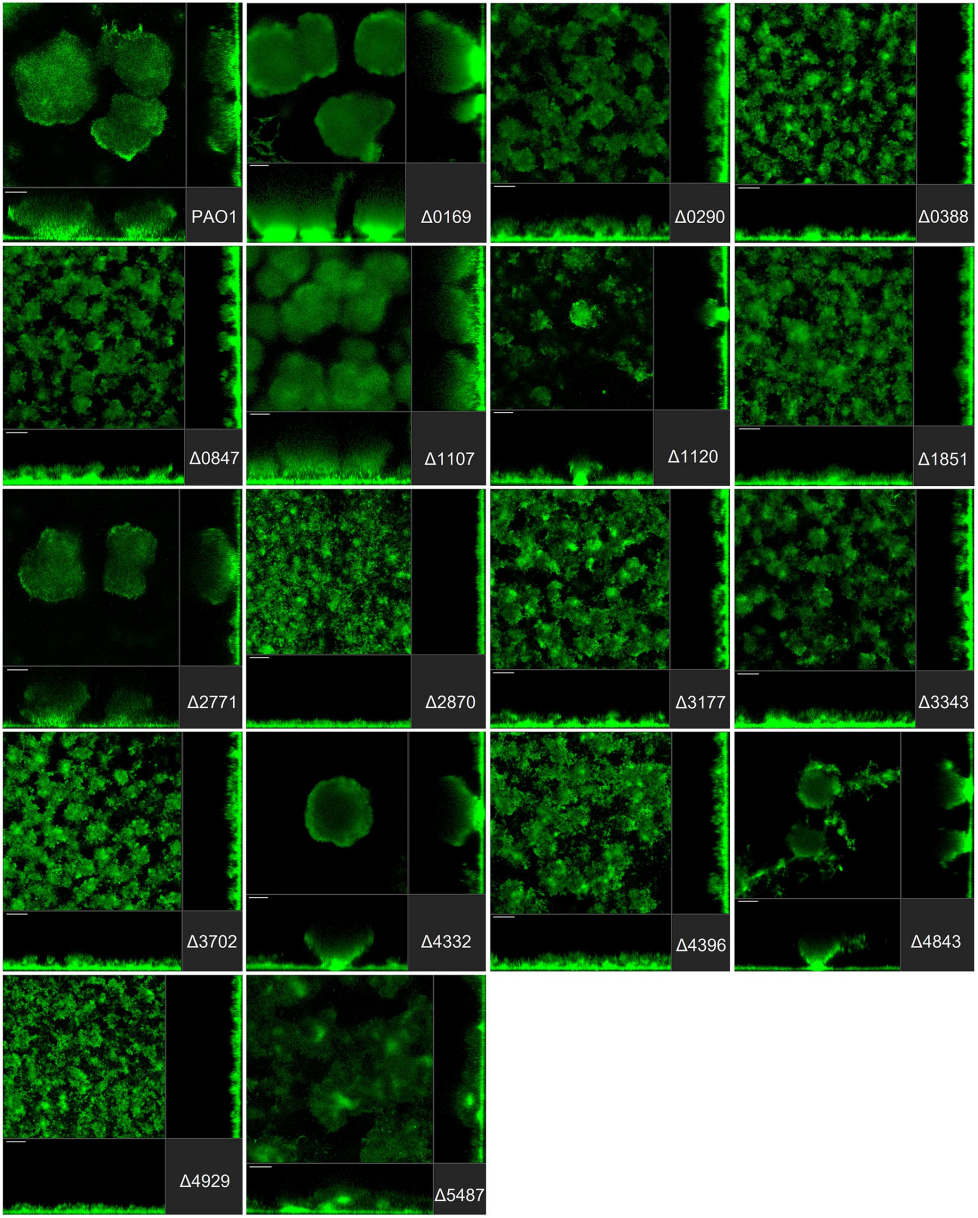
Impact of the deletion of genes encoding GGDEF proteins in *Pseudomonas aeruginosa* on biofilm structure characteristics. *P. aeruginosa* PAO1 WT and strains carrying a deletion of indicated c-di-GMP-metabolizing genes encoding GGDEF domain-containing enzymes were assessed for formation of biofilms by confocal scanning laser microscopy. GFP-tagged PAO1 WT and mutant strains were grown for 72 h in biofilm flow cell chambers under continuous feeding of ABTG medium. Confocal images were taken (three channels; seven images per channel). A representative image for each strain is shown.

**Figure 4:**
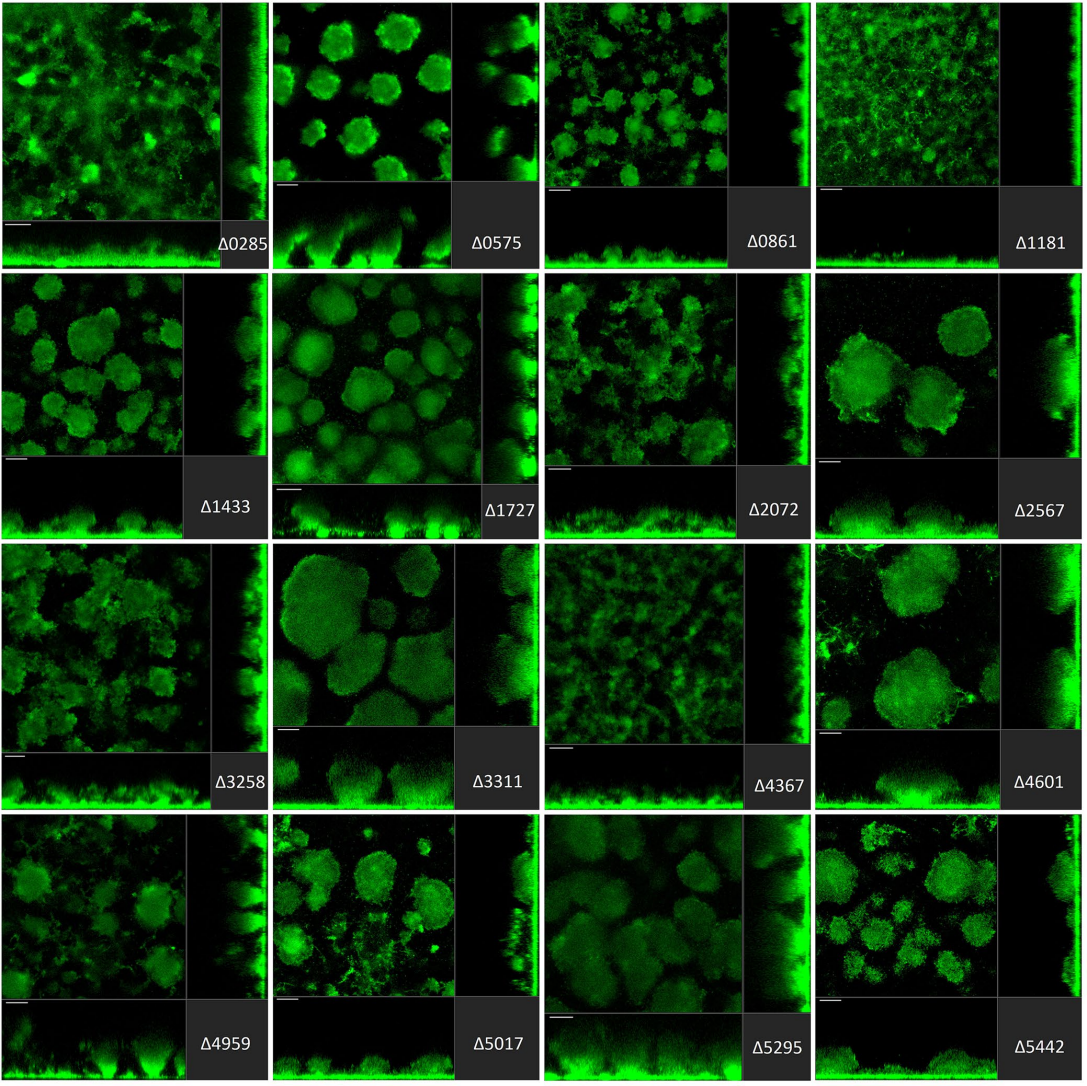
Impact of the deletion of genes encoding GGDEF/EAL dual proteins in *Pseudomonas aeruginosa* on biofilm structure characteristics. *P. aeruginosa* PAO1 WT and strains carrying a deletion of indicated c-di-GMP-metabolizing genes encoding GGDEF/EAL dual domain-containing enzymes were assessed for formation of biofilms by confocal scanning laser microscopy. GFP-tagged PAO1 WT and mutant strains were grown for 72 h in biofilm flow cell chambers under continuous feeding of ABTG medium. Confocal images were taken (three channels; seven images per channel). A representative image for each strain is shown.

**Figure 5:**
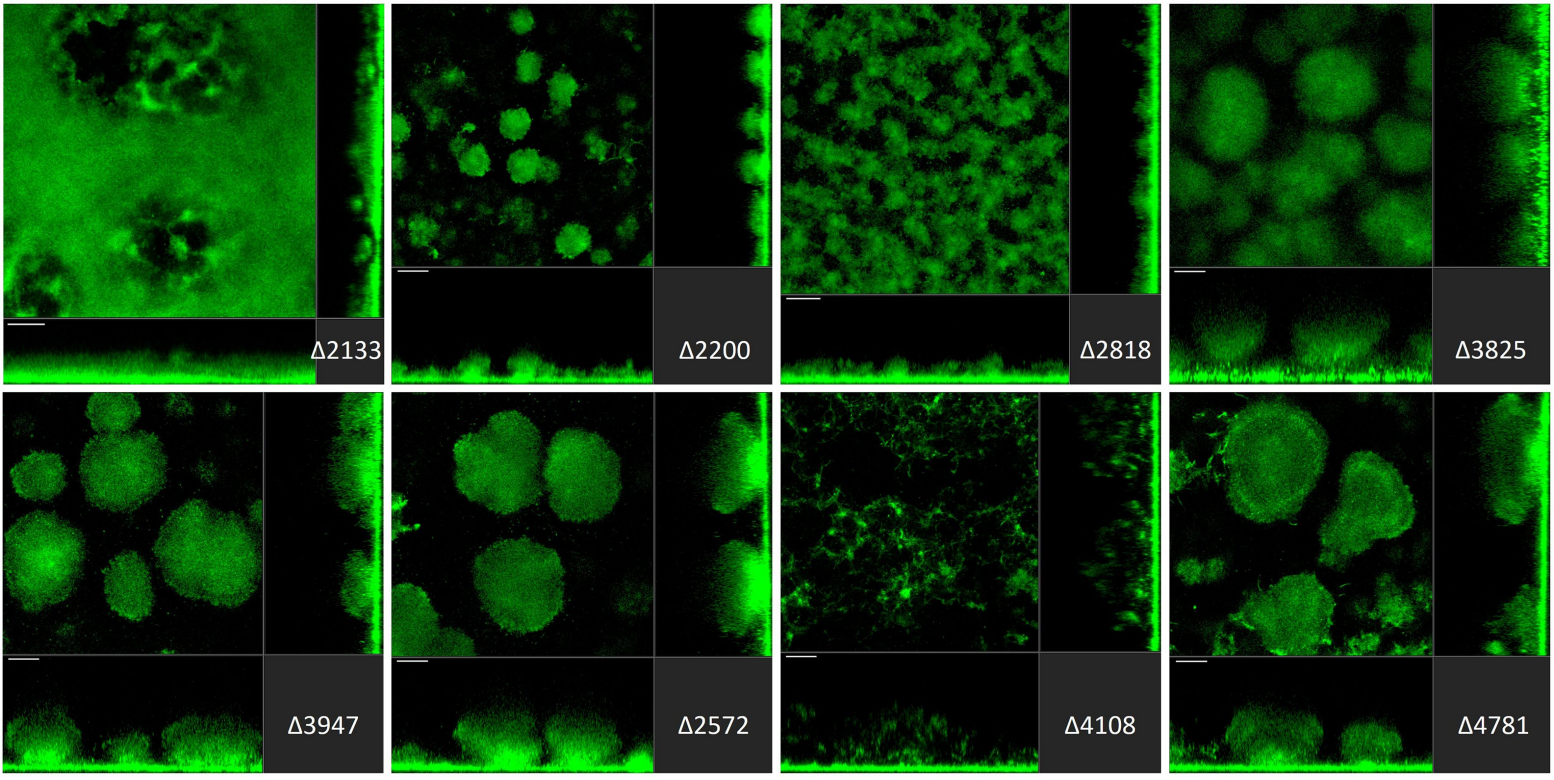
Impact of deletion of genes encoding EAL or HD-GYP proteins in *Pseudomonas aeruginosa* on biofilm structure characteristics. *P. aeruginosa* PAO1 WT and strains carrying a deletion of indicated c-di-GMP-metabolizing genes encoding EAL or HD-GYP domain-containing enzymes were assessed for formation of biofilms by confocal scanning laser microscopy. GFP-tagged PAO1 WT and mutant strains were grown for 72 h in biofilm flow cell chambers under continuous feeding of ABTG medium. Confocal images were taken (three channels; seven images per channel). A representative image for each strain is shown.

In the biofilm life cycle, once *P. aeruginosa* is committed to the surface, it can develop micro- and macro-colonies and evolve into a mature tri-dimensional biofilm architecture (O’Toole et al., 2000; Sauer et al., 2002). Several studies report that disrupting c-di-GMP homeostasis leads to altered *P. aeruginosa* biofilm structure. It is also important to recall that from initial attachment to fully mature biofilm, different regulatory determinants and different PDEs/DGCs may be required at any one stage (Valentini and Filloux, 2016). For this reason, it was important to assess not only the initial stage of biofilm formation as described above but also the maturation stage. Here, we subjected each individual strain of our mutant library to flow cell biofilm experiments and visualization using scanning confocal fluorescence microscopy. To this end, all strains in the library were engineered to constitutively express sf-GFP (Figueroa-Cuilan et al., 2016; Figures 3–5).

*Pseudomonas aeruginosa* PAO1 develops characteristic structures, notably by forming mushroom-like protrusions that punctuate the surface approximately 50 μm apart from each other (Figure 3A). Only a very thin lawn of cells covered the entire surface (side view Figure 3A). Strikingly, out of 41 mutants tested, more than half (22 mutants) showed alteration of the distinctive morphological mushroom-like biofilm shape, whereas 11 mutants differed in the frequency or size of the mushrooms (Figures 3–5). That 33 individual genes have a significant impact on the biofilm structure suggests that there are numerous signaling routes and molecular determinants of biofilm formation. Deletion of the genes encoding most GGDEF-containing DGCs (12 out of 17) resulted in the failure to develop mushroom shape formations (Figure 3). Instead, cells covered more of the total surface area in a lawn-like formation. Deletion of PA0169 (*siaD*; Klebensberger et al., 2009; Chen and Liang, 2020), PA1107 (*roeA*; Merritt et al., 2010) and PA4332 (*sadC*), caused the appearance of larger mushroom structures, whereas PA0290, PA1120 (*yfiN*) and PA4843 (*gcbA*) halted their development resulting in smaller structures. The deletion of PA2771 (*dcsbis*) had no visible impact on biofilm morphology. It is important to note that deletion of some of the genes that had no impact on biomass measured in attachment assays (Figure 2) nevertheless disrupted biofilm architecture profoundly (Figure 3). This suggests that there is a clear distinction in the role that GGDEF-containing enzymes may play at different stages during biofilm development. For example, it is remarkable that PA4332 (SadC) seems to be important for initial attachment with a drastic reduction for the *sadC* mutant, whereas this same mutant forms normal mushrooms, even bigger, after 72 h biofilm growth. This agrees with previous reports on SadC having its main role in the early stages of biofilm formation. However, PA0169 (SiaD), which has been reported to influence later biofilm development stages, was important for initial attachment, but not biofilm maturation in our study. This again reinforces the idea that comparison between studies might be challenging and an overall integration of the c-di-GMP network needs comprehensive and systematic studies using one single strain as reported here.

The deletion of GGDEF/EAL dual domain-encoding genes resulted in the complete disruption of mushroom-like formations in 6 out of 16 cases (Figure 4), including PA0285, PA0861 (*rbdA*), PA1181, PA2072, PA3258, PA4367 (*bifA*). Deletion of PA0575, PA1433 (*lapD*), PA1727 (*mucR*; Wang et al., 2015; Bellini et al., 2017; Zemke et al., 2020), PA4959 (*fimX*), PA5017 (*dipA*) and PA5442 had a far less significant impact but led to formation of smaller structures compared to PAO1. The absence of PA2567, PA3311, PA4601 (*morA*) and PA5295 (*proE*; Feng et al., 2020) did not cause any major changes in biofilm profiles. Here again, one notices that some genes that are significantly involved in initial attachment, e.g., PA2567, are of no importance for maturation of the biofilm after 72 h. Also, genes that are important for initial attachment, e.g., PA0285 and PA2567, may subsequently have quite different impacts on maturation by, respectively, failing to form mushrooms or not. This may suggest that both proteins have PDE activity at the initial stages of development, but that subsequently the PDE activity of PA2567 is turned off, or its putative DGC activity is triggered, and biofilm development is resumed.

Finally, among genes encoding EAL-containing PDEs, deletion of PA2133 (*fcsR*; Rossello et al., 2017), PA2200 and PA2818 resulted in the disruption of mushroom-like biofilm structures (Figure 5). Notably, the deletion mutant ∆PA2133 had the most severe impact on biofilm formation structures observed in the whole mutant library, resulting in a completely flat layer of cells, evenly distributed on the surface without any protruding structures (Figure 5). Interestingly, deletion of PA2133 had no impact on early biofilm attachment (Figure 2). As for the three genes encoding an HD-GYP domain-containing protein, only deletion of PA4108 resulted in the remodeling of biofilm architecture, leading to a thin layer of cells (Figure 5). This is consistent with the observation that the PA4108 mutant shows reduced attachment (Figure 2).

**Figure 6:**
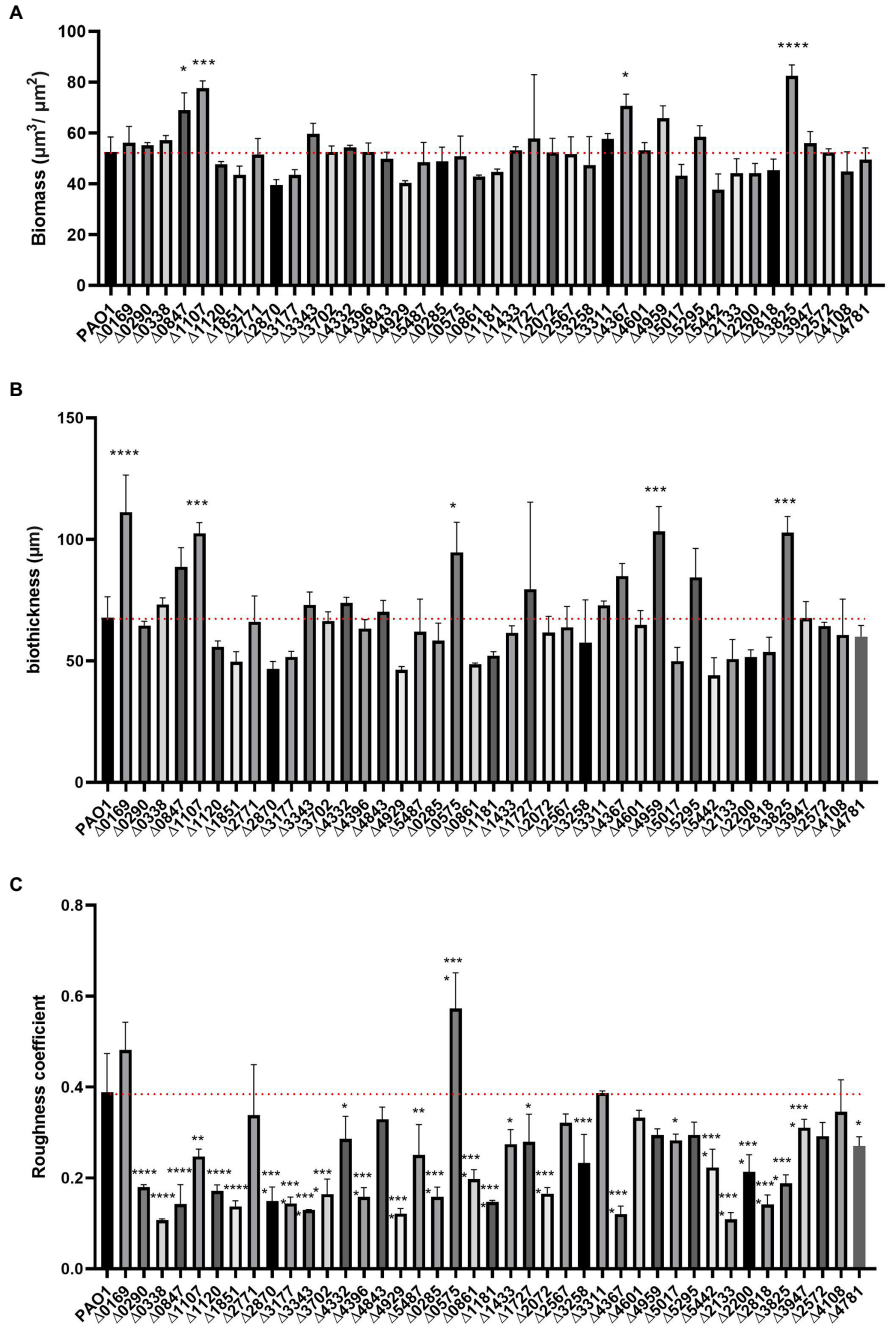
COMSTAT2 analysis of biofilm parameters of the c-di-GMP mutant library. Biofilm structure parameters, biomass **(A)**, biofilm thickness **(B)** and roughness coefficient - a measure of how much biofilm thickness varies **(C)** were analysed using the biofilm quantification algorithm COMSTAT2. Analysis was performed in biological triplicates. Statistical analysis was performed using One-way ANOVA, Tukey’s multiple comparison test (Student’s *t*-test, ^*^p < 0.05; ^**^p < 0.01; ^***^p < 0.001; ^****^p < 0.0001).

The confocal images of the developed biofilm structures were analyzed using the COMSTAT2 software (Heydorn et al., 2000) which supported the qualitative/quantitative comparison described above. Parameters tested were biomass, average thickness, and roughness coefficient. Each parameter describes a different aspect of the biofilm constitution. The biomass (volume per unit) is a measure of how much of the image stack is covered by bacteria, whereas the thickness quantifies the width of the biofilm over the stack of images and the dimensionless roughness coefficient Ra describes the roughness of a given surface (i.e., the mushroom aspect of the biofilm). The smaller the Ra value, the greater the uniformity of the biofilm. Biomass, thickness and roughness coefficient for each individual mutant strain are presented in Figure 6.

The biomass of the 41 deletion mutants did not differ significantly from the biomass determined for the PAO1 biofilm. Only four mutants exhibited elevated biomass measurements, namely PA0847 (Zhang et al., 2019), PA1107 (*roeA*; Cole and Lee, 2016), PA4367 and PA3825 (Figure 6A). This may be correlated to the observation that the PA1107 (Figure 3) mutant had an increase mushroom structure while PA3825 and PA4367 had an increased attachment rate (Figure 2). Yet, there are many other mutants showing these traits that do not have an overall change in biomass. This indicates that while the distribution of cells attached to the surface changes upon deletion of c-di-GMP-related genes, including remodeling of protruding mushroom-like structures from flat cell lawns, the overall attached cell mass does not change in most cases. As such, many mutants exhibiting retarded early attachment (biomass measured after 6 h, Figure 2) did manage, under continuous flow conditions for 72 h, to build a biofilm structure that did not differ from PAO1 in terms of biomass (Figure 6A).

Analysis of biofilm thickness resulted in a similar picture to the biomass findings. Only 5 mutants caused a significant upregulation of biofilm width compared to PAO1, while none showed a decrease in biofilm thickness (Figure 6B). Deletion of PA0169 (*siaD*), PA1107 (*roeA*), PA0575 (*rmcA*; Katharios-Lanwermeyer et al., 2021), PA4959 (*fimX*) and PA3825 generated an increase in measured biofilm thickness, which goes hand in hand with the increase in mushroom height (Figures 3–5). Analysis of the roughness coefficient, reflecting the uniformity of the biofilm (Figure 6C), revealed that mutation of most c-di-GMP-related genes resulted in a fundamental change and such changes could only be identified using confocal microscopy approaches as performed here. Only 10 of the 41 mutants (PA0169 (*siaD*), PA2771 (*dcsbis*), PA2567, PA4843 (*gcbA*), PA3311, PA4601 (*morA*), PA4959 (*fimX*), PA5295, PA2572, and PA4108) did not show a significant change in roughness coefficient. Overall, our analysis of the biofilm structure showed that all individual genes involved in c-di-GMP metabolism in PAO1 have an impact, and as such it suggests that there are many routes and signaling paths that are fine-tuning a so-called programmed development of the biofilm cycle.

### The absence of any single c-di-GMP-related gene barely affects bacterial motility in PAO1

The biofilm to motile switch has been reported in many instances with the underlying correlation that low levels of c-di-GMP promote motility and high c-di-GMP concentrations favor sessile bacteria. Here, we tested our library for two types of *P. aeruginosa* motility, swimming and twitching, which require a polar flagellum and type IV pili (T4P) respectively.

**Figure 7:**
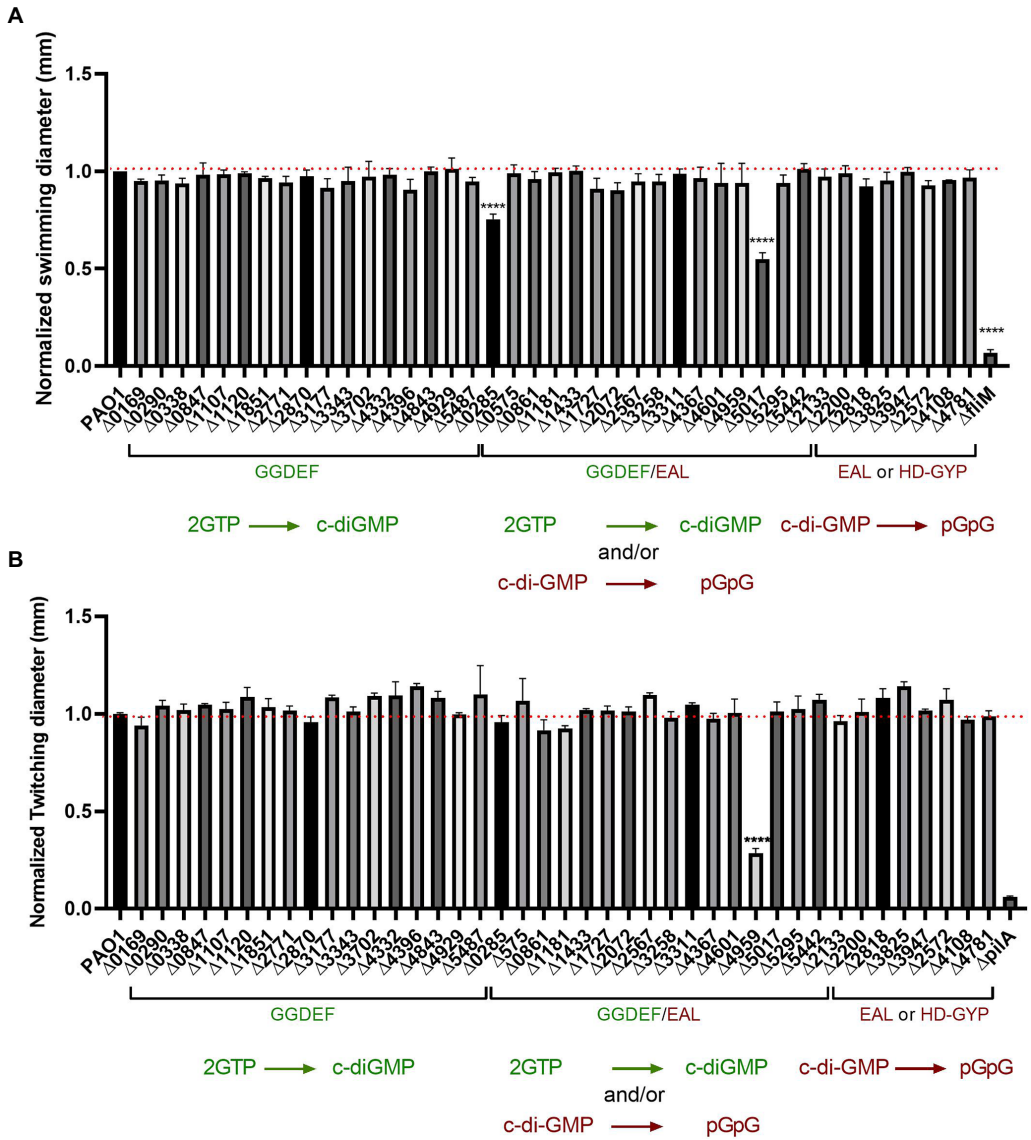
Impairment of c-di-GMP metabolizing genes produced almost no differences in bacterial motility. **(A)** Swimming motility of PAO1 WT and deletion mutant strains with indicated genes knocked out was assessed. Strains were grown overnight in LB broth at 37°C and injected straight from overnight cultures (0.5 μl) into the middle of swimming agar plates (0.2% agar) and incubated at 37°C for 16 h. Swimming diameter was measured. Assay was performed in biological triplicates and in 3 technical replicates and data normalized to PAO1 WT swimming diameter. (Student’s *t*-test, ^****^p < 0.0001). **(B)** Twitching motility was assessed for PAO1 WT and the indicated deletion mutants. Strains were grown on plate and picked up with the tip of a 200 μl pipette and inoculated on twitching plates (1% agar) for 48 h at 37°. Twitching diameter was measured and plotted. Assay was performed in three biological and technical replicates and data normalized to PAO1 WT twitching diameter. (Student’s *t*-test, ^****^p < 0.0001).

To test the flagellum-guided swimming motility, we conducted the assay in low percentage agar. The mutants were grown overnight in LB medium and 0.5 μl of undiluted culture was placed into the center of 0.2% agar plates, which were then incubated at 37°C overnight. Each mutant was tested in technical and biological triplicates and the diameter of the swimming zone was measured and normalized to PAO1, while the *fliM* mutant was used as a negative swimmer control (Barken et al., 2008). Results presented in Figure 7A showed almost no changes in swimming motility. Out of the 41 mutants assessed, only the deletion of two genes, PA0285 and PA5017 (*dipA*) showed a significant decrease in swimming motility. Both PA0285 and *dipA* mutants show elevated attachment profiles (Figure 2), which would therefore agree with the conceptual motile-to-sessile transition. It was previously described that *dipA* is important for dispersal and swarming motility, whereas there are no reports for PA0285.

To dissect contribution to T4P-driven motility behavior, the strains were streaked out on LB agar, grown overnight and subsequently picked up with a tip and stabbed to the bottom of a 1% LB agar plate. After growth of 48 h at 37°C, the diameter of the zone of twitching motility was measured and compared with PAO1; the *pilA* mutant was used as negative control (Koga et al., 1993). Results presented in Figure 7B strikingly show that only one of the 41 mutants tested had a significant reduction in twitching, i.e., PA4959 (*fimX*). FimX is a degenerated dual GGDEF/EAL domain-containing protein that binds c-di-GMP and has been previously confirmed to promote twitching motility by directly interacting with the T4P assembly ATPase, PilB (Jain et al., 2017).

Overall, we observed very limited impact on motility in our PAO1 mutant library. One might have expected that several other mutants would have been impaired as was the case in studies using the PA14 strain. This observation reinforces the need to compare c-di-GMP networks within the same individual parental strain.

### Limited variation in the global pool of c-di-GMP is observed in the c-di-GMP-related genes mutant library

**Figure 8:**
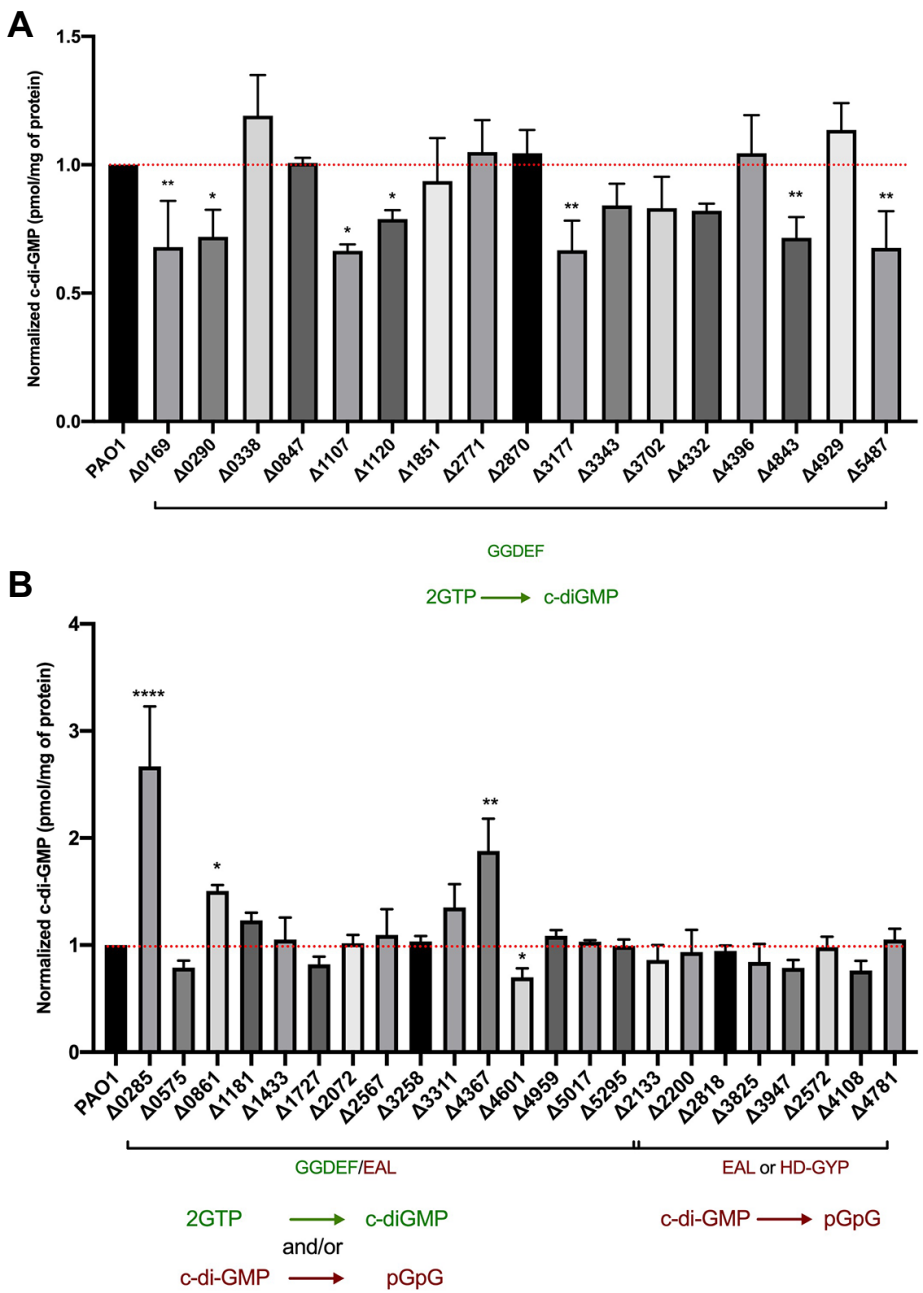
Assessment of intracellular c-di-GMP levels upon deletion of individual genes encoding DGCs and PDEs. LC–MS/MS quantification of c-di-GMP levels in *Pseudomonas aeruginosa* PAO1 WT and indicated mutant strains containing deletions of GGDEF **(A)** or dual GGDEF/EAL, or individual EAL or HD-GYP encoding genes **(B)**. Data are depicted as pmol c-di-GMP/mg of total protein and were normalized to PAO1 WT c-di-GMP levels. Each value is the average of two different biological replicates. (Student’s *t*-test, ^*^p < 0.05; ^**^p < 0.01; ^****^p < 0.0001).

After phenotypically assessing all 41 mutants in c-di-GMP-related phenotypes, we addressed the underlying hypothesis that deletion of putative *dgc/pde* genes would result in altered cellular c-di-GMP concentrations. To measure intracellular c-di-GMP levels, the wild-type and mutant strains were grown to stationary phase (6 h) under shaking conditions and c-di-GMP was extracted as described in Materials and Methods. Samples were compared to a standard curve derived from measurements of known concentrations of pure c-di-GMP. The data were then normalized to the total protein content in the sample determined by a Bradford assay. For each strain, the extraction was performed in biological duplicates and LC–MS/MS measurements were repeated in technical duplicates. The c-di-GMP measurements obtained are presented as pmol c-di-GMP/mg of total protein in Figure 8.

Deletion of genes encoding proteins solely containing a GGDEF domain led to the reduction of global c-di-GMP in 7 out of the 17 mutants (Figure 8A). Several of these proteins have been experimentally validated as active DGCs (Figure 1), including PA0169 (SiaD), PA1107 (RoeA), PA1120 (YfiN; Ueda and Wood, 2009), PA3177 (Poudyal and Sauer, 2018), PA4843 (GcbA) and PA5487 (DgcP). Previously studied GGDEF-containing enzymes that were shown to impact cellular concentrations of c-di-GMP, such as PA0847, PA2771 (Dcsbis), PA3702 (WspR; Hickman et al., 2005; De et al., 2010), PA4332 (SadC) and PA4929 (NicD; Basu Roy and Sauer, 2014), did not alter total c-di-GMP levels in our assay. Instead, the deletion of the PA0290 gene encoding an uncharacterized GGDEF-containing protein caused a significant reduction of c-di-GMP levels indicating its role as an active DGC.

When analyzing the impact of deletion of dual GGDEF/EAL harboring proteins, we noticed fewer changes in c-di-GMP levels. Out of 16 GGDEF/EAL dual domain-containing proteins, only the absence of 4 proteins, PA0285, PA0861 (RbdA), PA4367 (BifA), and PA4601 (MorA) led to a significant change in c-di-GMP levels. Notably, deletion of PA0285 led to the highest detected c-di-GMP concentration, increasing c-di-GMP by threefold compared to PAO1. Deletion of PA0285 also resulted in the highest detected increase in biofilm attachment (Figure 2) and was one of the two mutants that negatively impacted flagellum-mediated swimming motility (Figure 7A). Further, the deletion of PA0861 and PA4367 (*bifA*) caused a significant increase in c-di-GMP levels of 1.5- and 2-fold, respectively. These two mutants were also among those that displayed the strongest surface attachment (Figure 2). As for PA4601, c-di-GMP levels were reduced suggesting a DGC activity in the conditions of the assay. None of the mutants lacking an EAL- or HD-GYP-containing protein showed significant changes in c-di-GMP levels.

Overall, the variation in c-di-GMP level appears rather limited in most of the mutants, whereas phenotypic impact on attachment and biofilms is seen for most of them. This further suggests that c-di-GMP signaling might be effected from a localized subcellular compartment that does not necessarily reflect the global pool of the second messenger.

### Expression of c-di-GMP-related genes shows differences when comparing biofilm and planktonic growth conditions

Previous studies have shown that genes encoding c-di-GMP-metabolizing enzymes might be expressed differently under specific conditions, suggesting that some are readily expressed and might contribute to basal c-di-GMP levels (Wei et al., 2019), while expression of other genes is specifically induced under certain conditions to fine-tune specific phenotypic outputs. As such, the lack of phenotypic impact in some of our mutants could potentially be due to the corresponding gene not being expressed in our assay conditions. To assess the contribution of DGCs/PDEs to specific c-di-GMP-controlled phenotypes, we analyzed the transcript abundance of all 41 genes in both planktonic and biofilm states.

**Figure 9:**
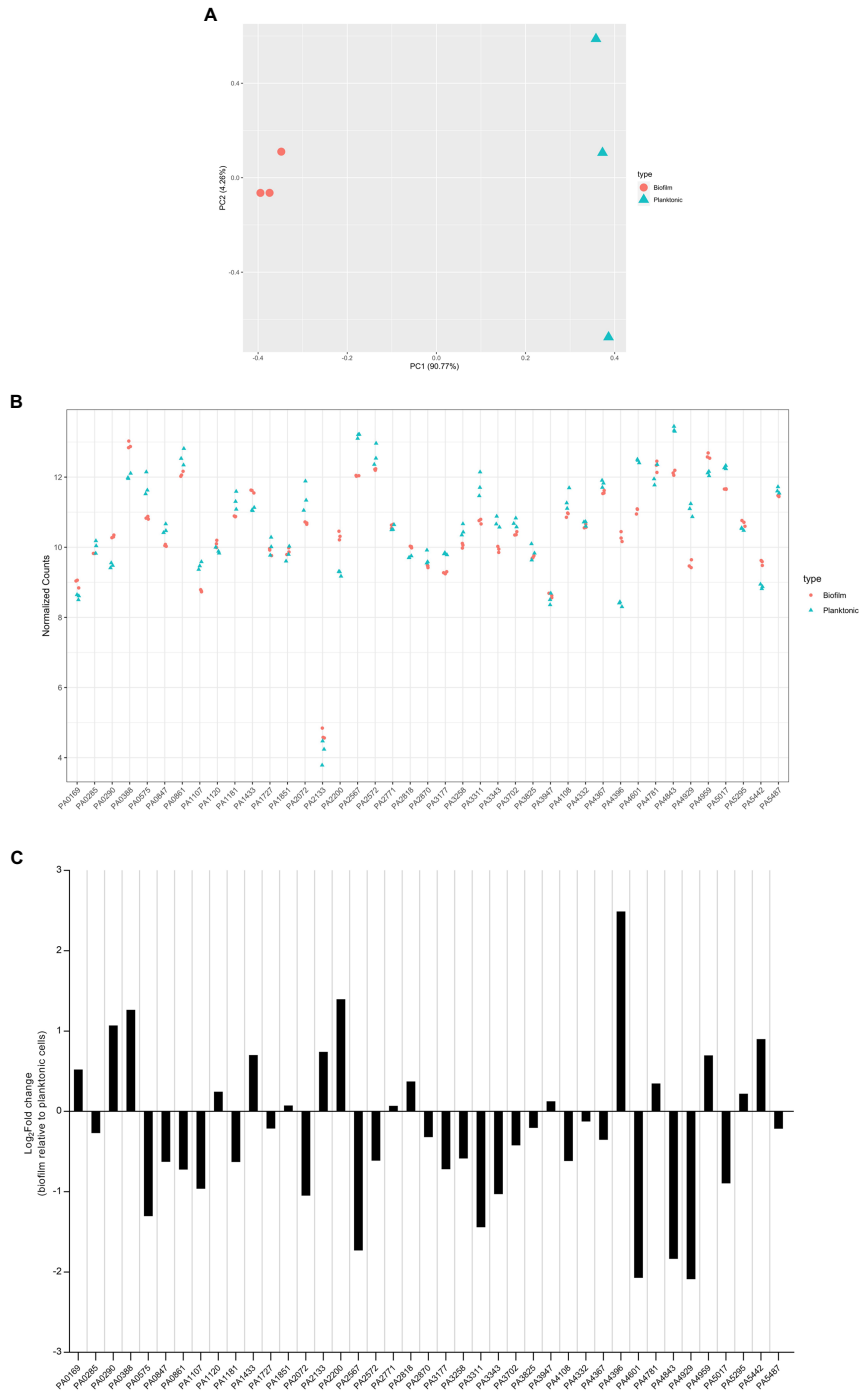
Expression level of *dgc/pde* genes in planktonic and biofilm growth. RNA-seq analysis was performed on total RNA extracted from *Pseudomonas aeruginosa* PAO1 WT grown either in planktonic conditions for 5 h at 37°C, under shaking (200 rpm) in ABTG medium or grown for 72 h in biofilm flow cell chambers under continuous feeding of ABTG medium. **(A)** Principal component analysis plot depicted the transcriptomes of *P. aeruginosa* PAO1 WT grown in either planktonic (triangle) or biofilm (circle) conditions. **(B)** RNA samples from planktonic (triangle) and biofilm (circle) conditions were prepared in triplicate from three independent biological samples and transcript abundance was analyzed and normalized using DESeq2 (Love et al., 2014) rlog transformation. **(C)** Log_2_Fold change of transcript abundance of genes encoding c-di-GMP modulating enzymes in biofilm relative to planktonic cells.

The transcriptomes of PAO1 cells grown under biofilm and planktonic conditions were distinct from each other (Figure 9A). Normalized transcript counts of all 41 c-di-GMP-metabolizing genes are presented as log_2_ fold change in Figure 9B. Except for PA2133, whose expression was very low, all genes were expressed at relatively high levels (Figure 9B). Further, the transcript abundance was analyzed in biofilm conditions relative to planktonic growth conditions. Interestingly, although there was not a big difference in normalized transcript counts in biofilm compared to planktonic growth (Figure 9C), it was apparent that some of the genes were more expressed in biofilm as compared to planktonic and vice versa. Interestingly, PA5017 which is more expressed in the planktonic state is one of the two genes whose mutation impacted *P. aeruginosa* swimming.

Our data indicate that all *dgc/pde* genes are expressed at a level which, if the gene is deleted, may have a significant phenotypic impact. That is clear from PA2133 which has the lowest expression but whose absence results in a mutant displaying a striking mushroom-less phenotype when analyzed with the flow cell set-up. It also shows that each *dgc/pde* seems to have a preferred context resulting in higher expression, either biofilm or planktonic, which suggests that appropriate phenotypes could be searched for taking this observation into account. Yet one should not consider that expression (mRNA levels) necessarily reflects function, since in many cases the DGC/PDE activity might need to be triggered by additional stimuli through the N-terminal sensing domains (Figure 1; Galperin et al., 2001).

## Discussion

Bacteria use the single messenger c-di-GMP to transmit information that results in multiple and distinct phenotypic outputs. The making and breaking of c-di-GMP involves two opposite enzymatic activities carried out by DGCs and PDEs, respectively. The understanding of complex c-di-GMP-regulated networks in bacteria such as *P. aeruginosa* that carry more than 40 *dgc/pde* genes is an important objective in the field. A growing body of evidence is building a narrative of complex modules of individual c-di-GMP genes, that upon deletion often lead to clear-cut phenotypes indicating that the multiplicity of *dgc/pde* genes does not mean redundancy.

**Figure 10:**
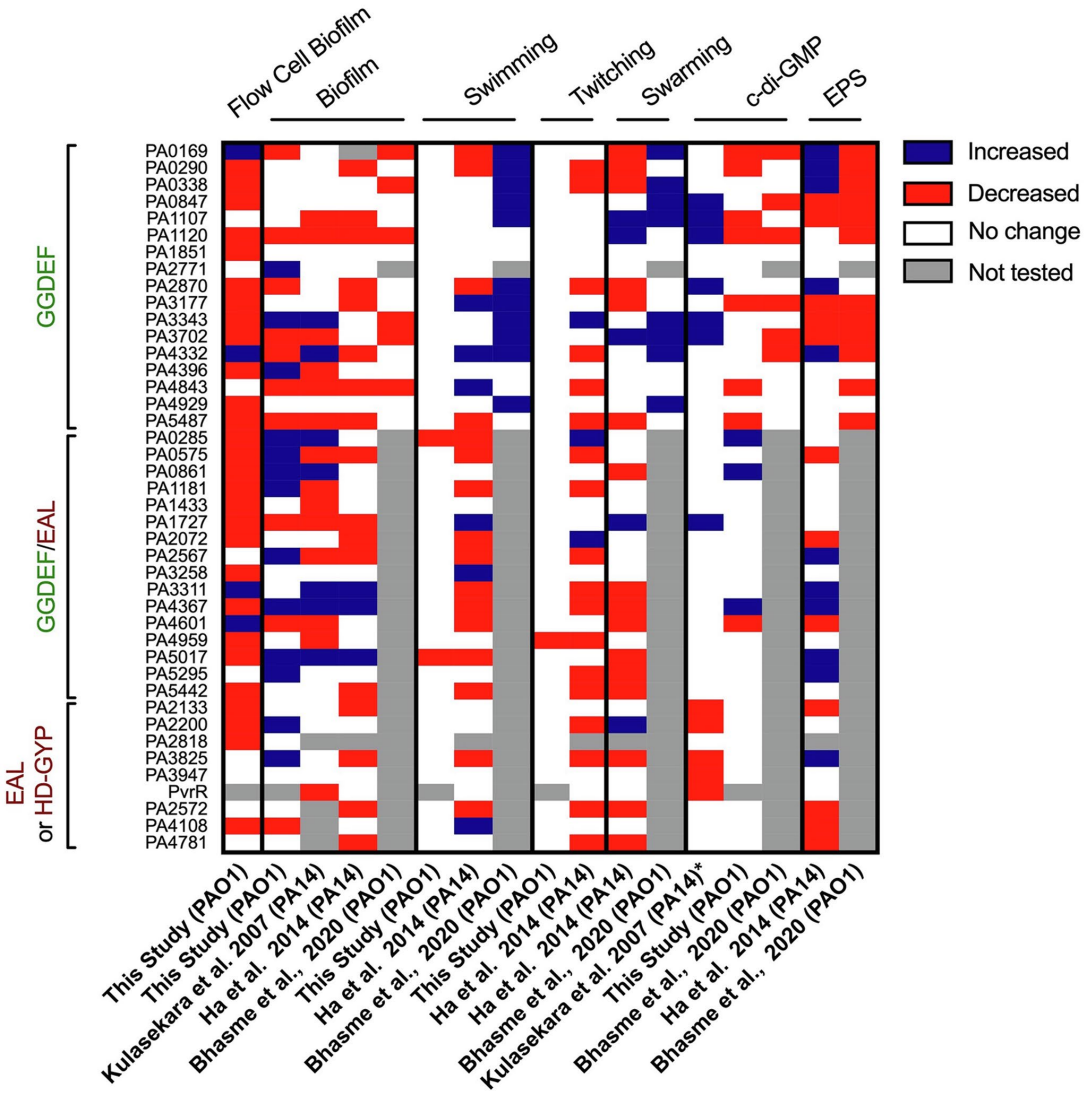
Heatmap overview comparing phenotypic screens using different *Pseudomonas aeruginosa* mutant libraries in c-di-GMP networks. The phenotypes assessed and corresponding study are indicated above and below, while the deleted genes are listed on the left. For each phenotype (biofilm, swimming, twitching, swarming, c-di-GMP level or EPS) an increase in activity/level is indicated in blue, a decrease in red, no change in phenotypic output in white and not tested or the absence of a mutant in gray. The study published by Kulasekara et al. screened a set of mutants of *P. aeruginosa* PA14 with transposon inserted in c-di-GMP-related genes (except from HD-GYP domain encoding genes) for their ability to form biofilm attachment and the enzymatic activity of encoded proteins (characterized by overexpression, *). The screen conducted by Ha et al. assessed all c-di-GMP genes in PA14 for swimming, twitching, swarming and EPS production. The study published by Bhasme et al. screens only genes encoding for a GGDEF domain and assessed phenotypes such as biofilm, swimming, swarming, EPS production and cellular c-di-GMP levels.

In our study, the systematic and individual deletion of all 41 *dgc/pde* genes revealed the “c-di-GMP fingerprint” of our *P. aeruginosa* “Lausanne” laboratory strain PAO1. While attachment and biofilm architecture phenotypes were significantly affected (Figures 2, 3), twitching and swimming motility were barely influenced, with only one and two mutants, respectively, showing a different phenotype (Figure 7). Biofilm formation involves dozens of distinct determinants from exopolysaccharides to extracellular appendages and cell surface adhesins, and therefore the fine-tuning of the selective expression of corresponding genes might require different paths and different *dgc/pde* genes. This is less likely to be the case for swimming and twitching motility that involve only flagella or T4P, respectively. Yet previous studies of c-di-GMP networks in PA14 (Kulasakara et al., 2006; Ha et al., 2014) or another PAO1 strain (Bhasme et al., 2020) have shown a drastic effect on motility-related behaviors (Figure 10). This re-emphasizes the importance of deciphering the impact that individual members of c-di-GMP networks may have in different *P. aeruginosa* strains, e.g., PA14 and PAO1. The differences observed between strains, beyond the genomic portfolio in *dgc/pde* genes, might be related to the fact that PAO1 and PA14 utilize different colonization strategies. While PAO1 tends to initially attach rapidly, increasing its surface-committed population quickly, PA14 uses a slower colonization approach. PAO1 utilizes a quick surge in Psl EPS, which is initiated by the Wsp surface-sensing system to rapidly attach to surfaces (Armbruster et al., 2019). PA14, in contrast, does not immediately attach, and slowly increases surface coverage. PA14 cells that already experienced contact with a surface get activated *via* the Pil-Chp system and a multigenerational cAMP memory, to form a surface-activated planktonic population that can later quickly attach and colonize surfaces (Lee et al., 2020). It is also noticeable that the LadS sensor, which promotes initial attachment through the Gac/Rsm pathway, is lacking in PA14 (Mikkelsen et al., 2011). Each of the Pil-Chp (Luo et al., 2015), Wsp (Armbruster et al., 2019) and Gac/Rsm (Moscoso et al., 2011) sensory systems have been linked to c-di-GMP circuits. Even when comparing the phenotypic impact of PA14 transposon or deletion mutant libraries, one can notice heterogeneity in the output. For example, the PA0861 (*rbdA*) mutant shows one of the largest increases in biofilm formation when looking at the transposon mutant (Kulasakara et al., 2006; Ha et al., 2014), while the clean deletion mutant shows barely any difference as compared with the parental strain (Ha et al., 2014; Figure 10). The same holds true with the PA4332 (*sadC*) mutant which is an hyper biofilm former in one case and a poor biofilm former in the other. However, the PA5017 (*dipA*) mutant is an hyperbiofilm former in both studies. It is important to note that in the study with clean deletion mutants the biofilm-related attachment assay was conducted using polystyrene multi-well plates instead of the glass used in the transposon mutant study, and a minimal medium was used instead of LB.

The contribution of individual c-di-GMP-metabolizing enzymes to overall c-di-GMP homeostasis, by determining the concentration in response to specific “environmental” conditions, is also an ongoing subject of investigation. We collected a coherent data set, measuring whole cell c-di-GMP levels upon deletion of each one of 41 c-di-GMP-metabolizing genes, using a mass-spectrometry approach. The only other comprehensive studies of this type used indirect approaches, such as overexpression of *dgc/pde* genes (Kulasakara et al., 2006) coupled with HPLC or the *cdrA-gfp* reporter assay (Rybtke et al., 2012). Here, one of the most striking observations is that, whereas most of the 41 deletion mutants tested showed a distinctive phenotypic impact in early biofilm attachment and mature biofilm architecture (Figures 2, 3), they show rather limited changes in the overall c-di-GMP levels (Figure 8). It is possible that this difference is due to the fact that c-di-GMP was measured under planktonic growth conditions, but we have observed that all *dgc/pde* were equally expressed either in planktonic or biofilm growth (Figure 9). In particular, the deletion of genes encoding an EAL domain alone or in combination with a GGDEF domain resulted in minimal changes to overall c-di-GMP concentration, but highly significant differences in biofilm architecture (Figures 3, 4). For example, the deletion mutant of PA2133 (encoding an EAL domain) caused one of the most pronounced phenotypes in biofilm structure; disrupting the PAO1 mushroom biofilm architecture completely (Figure 5). However, elimination of PA2133 had no impact at all on c-di-GMP levels (Figure 8B), implicating a more complex mechanism of action than just an adjustment of global c-di-GMP concentration. Several recent studies suggest that c-di-GMP levels fluctuate within single cells and support the possibility that local c-di-GMP pools control local regulatory networks, which involve specific DGCs, PDEs and effector proteins. We believe that this phenomenon and in particular the fluctuations cannot be assessed by our global c-di-GMP measurements. More specific approaches, such as those utilizing a fluorescence resonance energy transfer (FRET)-based biosensor combined with microscopy, may allow monitoring of spatiotemporal c-di-GMP concentrations within single bacterial cells (Christen et al., 2019). It should also be taken into consideration that measuring c-di-GMP levels from within a population may not be representative of the individual cells, especially in the context of a biofilm, and, as such, global measurements will not reflect cell heterogeneity (Nair et al., 2017).

Our study provides a systematic analysis of the contribution of single DGCs and PDEs to lifestyle behaviors of *P. aeruginosa* PAO1 and we put forward that the data we generated should be compared with data collected from similar studies performed by other laboratories and with other strains (Figure 10). Some of our mutants did not show any impact in any of the phenotypes tested. For example, PA3311 (*nbdA*; Xin et al., 2019; Zemke et al., 2020) did not exhibit any differential lifestyle behavior but its function as a PDE was previously characterized in detail (Li et al., 2013). This highlights the difficulty of assigning specific physiological roles to individual enzymes under laboratory conditions, since specific stimuli might be needed to trigger their activity. Indeed, most GGDEF/EAL-containing proteins harbor additional accessory domains that act as sensor or receiver modules, and as such the input signal must be identified and associated with the various growth conditions tested. This is even more important since we showed that all the *dgc/pde* genes are significantly expressed in both planktonic and biofilm growth conditions as used for our assays. While stimuli such as light (Barends et al., 2009), oxygen (Tuckerman et al., 2009), nitric oxide (Barraud et al., 2006; Plate and Marletta, 2012), nutrients (Basu Roy and Sauer, 2014), metals (Zahringer et al., 2013), small molecules (Andersen et al., 2021) or surface contact (Laventie et al., 2019) have been identified to activate particular DGCs and PDEs, many enzymes have not been assigned an input signal yet, which may be due to the limited physiological conditions that are assayed in a laboratory setting. It also must be taken into consideration that more than one DGC or PDE can contribute to regulating a specific physiological process. The library of single c-di-GMP-metabolizing genes created in this study should thus be a starting point for further engineering combinatorial mutants in PAO1 and extend our understanding of the c-di-GMP network in this specific background.

## Data availability statement

The datasets presented in this study can be found in online repositories. The names of the repository/repositories and accession number(s) can be found in the article/Supplementary Material.

## Author contributions

KE: conceptualization, investigation, methodology, and writing—original draft. JK, RM, AM, JB, CH, and XL: investigation and methodology. MG, SR, and AF: conceptualization, funding acquisition, supervision, and writing—review and editing. All authors contributed to the article and approved the submitted version.

## Funding

KE was supported by a Wellcome Trust Scholarship and RM by a MRC Clinical Research Training Fellowship. AF work was supported by BBSRC grant BB/R00174X/1. The authors would like to acknowledge the financial support from National Research Foundation and Ministry of Education Singapore under its Research Centre of Excellence Program.

## Conflict of interest

The authors declare that the research was conducted in the absence of any commercial or financial relationships that could be construed as a potential conflict of interest.

## Publisher’s note

All claims expressed in this article are solely those of the authors and do not necessarily represent those of their affiliated organizations, or those of the publisher, the editors and the reviewers. Any product that may be evaluated in this article, or claim that may be made by its manufacturer, is not guaranteed or endorsed by the publisher.

## Footnotes

^1^ http://www.comstat.dk/

^2^ https://www.pseudomonas.com/

^3^ https://www.ncbi.nlm.nih.gov/Structure/cdd/wrpsb.cgi
