# Supplementary material for: Phenotypic and integrated analysis of a comprehensive Pseudomonas aeruginosa PAO1 library of mutants lacking cyclic-di-GMP-related genes
Source: Front Microbiol. 2022 Jul 22;13:949597. doi: 10.3389/fmicb.2022.949597 (PMC9355167; doi:10.3389/fmicb.2022.949597)
Supplement: Supplementary file 1 [file Table_1.docx]

**SUPPLEMENTARY TABLES 1-3**

**Table 1. Strains used in this study**

| **Strain** | **Relevant features** | **Reference** |
| --- | --- | --- |
| DH5a | Strain used for cloning and maintenance of plasmids. F^–^*endA1* *glnV44* *thi*^-^. *recA1* *relA1 gyrA96* *deoR nupG purB20* φ80d*lacZ*ΔM15 Δ(*lacZYA*-*argF*)U169, *hsdR17*(r*_K_*^-^m*_K_*^+^ ),λ^-^. | Invitrogen |
| CC118λpir | Strain used for pKNG101 maintenance. Δ(*ara-leu*) *araD* Δ*lacX74* *galE galK-phoA20* *thi-1 rpsE rpoB argE (*ApR*) recA1 Rfr λpir.* | Laboratory  collection |
| PAO1 | Wild-type strain, parental used for all experiments, Lausanne subline | Dieter Haas |
| PAO1∆PA0169 | Deletion of PA0169 in PAO1 | This study |
| PAO1∆PA0285 | Deletion of PA0285 in PAO1 | This study |
| PAO1∆PA0290 | Deletion of PA0290 in PAO1 | This study |
| PAO1∆PA0338 | Deletion of PA0338 in PAO1 | This study |
| PAO1∆PA0575 | Deletion of PA0575 in PAO1 | This study |
| PAO1∆PA0847 | Deletion of PA0847 in PAO1 | This study |
| PAO1∆PA0861 | Deletion of PA0861 in PAO1 | This study |
| PAO1∆PA1107 | Deletion of PA1107 in PAO1 | This study |
| PAO1∆PA1120 | Deletion of PA1120 in PAO1 | This study |
| PAO1∆PA1181 | Deletion of PA1181 in PAO1 | This study |
| PAO1∆PA1433 | Deletion of PA1433 in PAO1 | This study |
| PAO1∆PA1727 | Deletion of PA1727 in PAO1 | This study |
| PAO1∆PA1851 | Deletion of PA1851 in PAO1 | This study |
| PAO1∆PA2072 | Deletion of PA2071 in PAO1 | This study |
| PAO1∆PA2133 | Deletion of PA2133 in PA01 | This study |
| PAO1∆PA2200 | Deletion of PA2200 in PAO1 | This study |
| PAO1∆PA2567 | Deletion of PA2567 in PAO1 | This study |
| PAO1∆PA2572 | Deletion of PA2572 in PAO1 | This study |
| PAO1∆PA2771 | Deletion of PA2771 in PAO1 | This study |
| PAO1∆PA2818 | Deletion of PA2818 in PAO1 | This study |
| PAO1∆PA2870 | Deletion of PA2870 in PAO1 | This study |
| PAO1∆PA3177 | Deletion of PA3177 in PAO1 | This study |
| PAO1∆PA3258 | Deletion of PA3258 in PAO1 | This study |
| PAO1∆PA3311 | Deletion of PA3311 in PAO1 | This study |
| PAO1∆PA3343 | Deletion of PA3343 in PAO1 | This study |
| PAO1∆PA3701 | Deletion of PA3702 in PAO1 | This study |
| PAO1∆PA3825 | Deletion of PA3825 in PAO1 | This study |
| PAO1∆PA3947 | Deletion of PA3947 in PAO1 | This study |
| PAO1∆PA4108 | Deletion of PA4108 in PAO1 | This study |
| PAO1∆PA4332 | Deletion of PA4332 in PAO1 | This study |
| PAO1∆PA4367 | Deletion of PA4367 in PAO1 | This study |
| PAO1∆PA4396 | Deletion of PA4396 in PAO1 | This study |
| PAO1∆PA4601 | Deletion of PA4601 in PAO1 | This study |
| PAO1∆PA4781 | Deletion of PA4781 in PAO1 | This study |
| PAO1∆PA4843 | Deletion of PA4843 in PAO1 | This study |
| PAO1∆PA4929 | Deletion of PA4929 in PAO1 | This study |
| PAO1∆PA4959 | Deletion of PA4959 in PAO1 | This study |
| PAO1∆PA5017 | Deletion of PA5017 in PAO1 | This study |
| PAO1∆PA5295 | Deletion of PA5295 in PAO1 | This study |
| PAO1∆PA5442 | Deletion of PA5442 in PAO1 | This study |
| PAO1∆PA5487 | Deletion of PA5487 in PAO1 | This study |
| PAO1mini-Tn7-*egfp* | Wild-type strain, mini-Tn7-*egfp* integrated at attTn7 site, GmR | Laboratory  collection |
| PAO1∆PA0169 mini-Tn7-*egfp* | Deletion of PA0169 in PAO1, mini-Tn7-*egfp* integrated at attTn7 site, GmR | This study |
| PAO1∆PA0285 mini-Tn7-*egfp* | Deletion of PA0285 in PAO1, mini-Tn7-*egfp* integrated at attTn7 site, GmR | This study |
| PAO1∆PA0290 mini-Tn7-*egfp* | Deletion of PA0290 in PAO1, mini-Tn7-*egfp* integrated at attTn7 site, GmR | This study |
| PAO1∆PA0338 mini-Tn7-*egfp* | Deletion of PA0338 in PAO1, mini-Tn7-*egfp* integrated at attTn7 site, GmR | This study |
| PAO1∆PA0575 mini-Tn7-*egfp* | Deletion of PA0575 in PAO1, mini-Tn7-*egfp* integrated at attTn7 site, GmR | This study |
| PAO1∆PA0847 mini-Tn7-*egfp* | Deletion of PA0847 in PAO1, mini-Tn7-*egfp* integrated at attTn7 site, GmR | This study |
| PAO1∆PA0861 mini-Tn7-*egfp* | Deletion of PA0861 in PAO1, mini-Tn7-*egfp* integrated at attTn7 site, GmR | This study |
| PAO1∆PA1107 mini-Tn7-*egfp* | Deletion of PA1107 in PAO1, mini-Tn7-*egfp* integrated at attTn7 site, GmR | This study |
| PAO1∆PA1120 mini-Tn7-*egfp* | Deletion of PA1120 in PAO1, mini-Tn7-*egfp* integrated at attTn7 site, GmR | This study |
| PAO1∆PA1181 mini-Tn7-*egfp* | Deletion of PA1181 in PAO1, mini-Tn7-*egfp* integrated at attTn7 site, GmR | This study |
| PAO1∆PA1433 mini-Tn7-*egfp* | Deletion of PA1433 in PAO1, mini-Tn7-*egfp* integrated at attTn7 site, GmR | This study |
| PAO1∆PA1727 mini-Tn7-*egfp* | Deletion of PA1727 in PAO1, mini-Tn7-*egfp* integrated at attTn7 site, GmR | This study |
| PAO1∆PA1851 mini-Tn7-*egfp* | Deletion of PA1851 in PAO1, mini-Tn7-*egfp* integrated at attTn7 site, GmR | This study |
| PAO1∆PA2072 mini-Tn7-*egfp* | Deletion of PA2071 in PAO1, mini-Tn7-*egfp* integrated at attTn7 site, GmR | This study |
| PAO1∆PA2133 mini-Tn7-*egfp* | Deletion of PA2133 in PA01, mini-Tn7-*egfp* integrated at attTn7 site, GmR | This study |
| PAO1∆PA2200 mini-Tn7-*egfp* | Deletion of PA2200 in PAO1, mini-Tn7-*egfp* integrated at attTn7 site, GmR | This study |
| PAO1∆PA2567 mini-Tn7-*egfp* | Deletion of PA2567 in PAO1, mini-Tn7-*egfp* integrated at attTn7 site, GmR | This study |
| PAO1∆PA2572 mini-Tn7-*egfp* | Deletion of PA2572 in PAO1, mini-Tn7-*egfp* integrated at attTn7 site, GmR | This study |
| PAO1∆PA2771 mini-Tn7-*egfp* | Deletion of PA2771 in PAO1, mini-Tn7-*egfp* integrated at attTn7 site, GmR | This study |
| PAO1∆PA2818 mini-Tn7-*egfp* | Deletion of PA2818 in PAO1, mini-Tn7-*egfp* integrated at attTn7 site, GmR | This study |
| PAO1∆PA2870 mini-Tn7-*egfp* | Deletion of PA2870 in PAO1, mini-Tn7-*egfp* integrated at attTn7 site, GmR | This study |
| PAO1∆PA3177 mini-Tn7-*egfp* | Deletion of PA3177 in PAO1, mini-Tn7-*egfp* integrated at attTn7 site, GmR | This study |
| PAO1∆PA3258 mini-Tn7-*egfp* | Deletion of PA3258 in PAO1, mini-Tn7-*egfp* integrated at attTn7 site, GmR | This study |
| PAO1∆PA3311 mini-Tn7-*egfp* | Deletion of PA3311 in PAO1, mini-Tn7-*egfp* integrated at attTn7 site, GmR | This study |
| PAO1∆PA3343 mini-Tn7-*egfp* | Deletion of PA3343 in PAO1, mini-Tn7-*egfp* integrated at attTn7 site, GmR | This study |
| PAO1∆PA3701 mini-Tn7-*egfp* | Deletion of PA3702 in PAO1, mini-Tn7-*egfp* integrated at attTn7 site, GmR | This study |
| PAO1∆PA3825 mini-Tn7-*egfp* | Deletion of PA3825 in PAO1, mini-Tn7-*egfp* integrated at attTn7 site, GmR | This study |
| PAO1∆PA3947 mini-Tn7-*egfp* | Deletion of PA3947 in PAO1, mini-Tn7-*egfp* integrated at attTn7 site, GmR | This study |
| PAO1∆PA4108 mini-Tn7-*egfp* | Deletion of PA4108 in PAO1, mini-Tn7-*egfp* integrated at attTn7 site, GmR | This study |
| PAO1∆PA4332 mini-Tn7-*egfp* | Deletion of PA4332 in PAO1, mini-Tn7-*egfp* integrated at attTn7 site, GmR | This study |
| PAO1∆PA4367 mini-Tn7-*egfp* | Deletion of PA4367 in PAO1, mini-Tn7-*egfp* integrated at attTn7 site, GmR | This study |
| PAO1∆PA4396 mini-Tn7-*egfp* | Deletion of PA4396 in PAO1, mini-Tn7-*egfp* integrated at attTn7 site, GmR | This study |
| PAO1∆PA4601 mini-Tn7-*egfp* | Deletion of PA4601 in PAO1, mini-Tn7-*egfp* integrated at attTn7 site, GmR | This study |
| PAO1 ∆PA4781 mini-Tn7-egfp | Deletion of PA4781 in PAO1, mini-Tn7-*egfp* integrated at attTn7 site, GmR | This study |
| PAO1∆PA4843 mini-Tn7-*egfp* | Deletion of PA4843 in PAO1, mini-Tn7-*egfp* integrated at attTn7 site, GmR | This study |
| PAO1∆PA4929 mini-Tn7-*egfp* | Deletion of PA4929 in PAO1, mini-Tn7-*egfp* integrated at attTn7 site, GmR | This study |
| PAO1∆PA4959 mini-Tn7-*egfp* | Deletion of PA4959 in PAO1, mini-Tn7-*egfp* integrated at attTn7 site, GmR | This study |
| PAO1∆PA5017 mini-Tn7-*egfp* | Deletion of PA5017 in PAO1, mini-Tn7-*egfp* integrated at attTn7 site, GmR | This study |
| PAO1∆PA5295 mini-Tn7-*egfp* | Deletion of PA5295 in PAO1, mini-Tn7-*egfp* integrated at attTn7 site, GmR | This study |
| PAO1∆PA5442 mini-Tn7-*egfp* | Deletion of PA5442 in PAO1, mini-Tn7-*egfp* integrated at attTn7 site, GmR | This study |
| PAO1∆PA5487 mini-Tn7-*egfp* | Deletion of PA5487 in PAO1, mini-Tn7-*egfp* integrated at attTn7 site, GmR | This study |

**Table 2. Plasmids used in this study**

| **Plasmid** | **Characteristics** | **Source** |
| --- | --- | --- |
| pCRTM-Blunt II-TOPOTM | Cloning vector for constructs synthesized by KOD PCR, KmR | Invitrogen |
| pRK2013 | Helper plasmid for three- partner conjugations, KmR | Laboratory collection |
| pKNG101 | Non-replicative suicide vector for *P. aeruginosa* chromosome mutagenesis, *ori6K, mobRK2, sacB* gene for sucrose sensitivity, SmR | Laboratory collection |
| pKNG101∆PA0169 | Suicide vector to delete PA0169 from *P. aeruginosa*, SmR | This study |
| pKNG101∆PA0285 | Suicide vector to delete PA0285 from *P. aeruginosa*, SmR | This study |
| pKNG101∆PA0290 | Suicide vector to delete PA0290 from *P. aeruginosa*, SmR | This study |
| pKNG101∆PA0338 | Suicide vector to delete PA0388 from *P. aeruginosa*, SmR | This study |
| pKNG101∆PA0575 | Suicide vector to delete PA0575 from P. aeruginosa, SmR | This study |
| pKNG101∆PA0847 | Suicide vector to delete PA0847 from P. aeruginosa, SmR | This study |
| pKNG101∆PA0861 | Suicide vector to delete PA0861 from P. aeruginosa, SmR | This study |
| pKNG101∆PA1107 | Suicide vector to delete PA1107 from *P. aeruginosa*, SmR | This study |
| pKNG101∆PA1120 | Suicide vector to delete PA1120 from *P. aeruginosa*, SmR | This study |
| pKNG101∆PA1181 | Suicide vector to delete PA1181 from *P. aeruginosa*, SmR | This study |
| pKNG101∆PA1433 | Suicide vector to delete PA1433 from *P. aeruginosa*, SmR | This study |
| pKNG101∆PA1727 | Suicide vector to delete PA1727 from *P. aeruginosa*, SmR | This study |
| pKNG101∆PA1851 | Suicide vector to delete PA1851 from *P. aeruginosa*, SmR | This study |
| pKNG101∆PA2072 | Suicide vector to delete PA2072 from *P. aeruginosa*, SmR | This study |
| pKNG101∆PA2133 | Suicide vector to delete PA2133 from *P. aeruginosa*, SmR | This study |
| pKNG101∆PA2200 | Suicide vector to delete PA2200 from *P. aeruginosa*, SmR | This study |
| pKNG101∆PA2567 | Suicide vector to delete PA2567 from *P. aeruginosa*, SmR | This study |
| pKNG101∆PA2572 | Suicide vector to delete PA2572 from *P. aeruginosa*, SmR | This study |
| pKNG101∆PA2771 | Suicide vector to delete PA2771 from *P. aeruginosa*, SmR | This study |
| pKNG101∆PA2818 | Suicide vector to delete PA2818 from *P. aeruginosa*, SmR | This study |
| pKNG101∆PA2870 | Suicide vector to delete PA2870 from *P. aeruginosa*, SmR | This study |
| pKNG101∆PA3177 | Suicide vector to delete PA3177 from *P. aeruginosa*, SmR | This study |
| pKNG101∆PA3258 | Suicide vector to delete PA3258 from *P. aeruginosa*, SmR | This study |
| pKNG101∆PA3311 | Suicide vector to delete PA3311 from *P. aeruginosa*, SmR | This study |
| pKNG101∆PA3343 | Suicide vector to delete PA3343 from *P. aeruginosa*, SmR | This study |
| pKNG101∆PA3701 | Suicide vector to delete PA3701 from *P. aeruginosa*, SmR | This study |
| pKNG101∆PA3825 | Suicide vector to delete PA3825 from *P. aeruginosa*, SmR | This study |
| pKNG101∆PA3947 | Suicide vector to delete PA3947 from *P. aeruginosa*, SmR | This study |
| pKNG101∆PA4108 | Suicide vector to delete PA4108 from *P. aeruginosa*, SmR | This study |
| pKNG101∆PA4332 | Suicide vector to delete PA4332 from *P. aeruginosa*, SmR | This study |
| pKNG101∆PA4367 | Suicide vector to delete PA4367 from *P. aeruginosa*, SmR | This study |
| pKNG101∆PA4396 | Suicide vector to delete PA4396 from *P. aeruginosa*, SmR | This study |
| pKNG101∆PA4601 | Suicide vector to delete PA4601 from *P. aeruginosa*, SmR | This study |
| pKNG101∆PA4781 | Suicide vector to delete PA4781 from *P. aeruginosa*, SmR | This study |
| pKNG101∆PA4843 | Suicide vector to delete PA4843 from *P. aeruginosa*, SmR | This study |
| pKNG101∆PA4929 | Suicide vector to delete PA4929 from *P. aeruginosa*, SmR | This study |
| pKNG101∆PA4959 | Suicide vector to delete PA4959 from *P. aeruginosa*, SmR | This study |
| pKNG101∆PA5017 | Suicide vector to delete PA5017 from *P. aeruginosa*, SmR | This study |
| pKNG101∆PA5295 | Suicide vector to delete PA5295 from *P. aeruginosa*, SmR | This study |
| pKNG101∆PA5442 | Suicide vector to delete PA5442 from *P. aeruginosa*, SmR | This study |
| pKNG101∆PA5487 | Suicide vector to delete PA5487 from *P. aeruginosa*, SmR | This study |
| miniTn7::egfp | Broad host-range mini-Tn7 vector integration of single-copy genes at attTn7 | Laboratory collection |

**Table 3. Primers used in this study**

| **Name** | **Sequence** |
| --- | --- |
| ∆PA0169 P1 | CGCCGTGATCTGACCCCGTGT |
| ∆PA0169 P2 | GTCGAGCAGGTTCTGCTCCCGCGCCAT |
| ∆PA0169 P3 | GAGCAGAACCTGCTCGACGCCAAGCGC |
| ∆PA0169 P4 | CGGTGGCCGACGAGGACGAG |
| ∆PA0169 P5 | CCTGGTGGAACGCGACGACG |
| ∆PA0169 P6 | CCCGTGCACCTGATCTTCCTGC |
| ∆PA0285 P1 | ACGGTGATCTCGACGGCG |
| ∆PA0285 P2 | CGCCGCAAGCTGGCCATCGACGACTTC |
| ∆PA0285 P3 | GATGGCCAGCTTGCGGCGGAACTGGCG |
| ∆PA0285 P4 | ACGGTGGATAGCGGTCCA |
| ∆PA0285 P5 | CATTCGCGGCCGACAGCT |
| ∆PA0285 P6 | CACGGCCCAGACGGTCAG |
| ∆PA0290 P1 | CGACGCTGGGCGAGATCT |
| ∆PA0290 P2 | TCAGCCCACGTCGTCCATGGATCGTCG |
| ∆PA0290 P3 | ATGGACGACGTGGGCTGACCGCTGGGC |
| ∆PA0290 P4 | ATCAGGAAACGGCGCAACC |
| ∆PA0290 P5 | CTGTTTCGCCAGCGCGCC |
| ∆PA0290 P6 | GCAGGGTCAGCGATGCCTTG |
| ΔPA0338 P1 | GACTTCCTTTCGGTCGGTTC |
| ΔPA0338 P2 | ATCGCGACGCACGACGAAGCGTCC |
| ΔPA0338 P3 | TTCGTCGTGCGTCGCGATTGCGTG |
| ΔPA0338 P4 | CCATCAGCTCTGGCACTACA |
| ΔPA0338 P5 | ATCCTGCTGCCGATGATCT |
| ΔPA0338 P6 | ACTATTCCGGCTTCAACCTG |
| ∆PA0575 P1 | CATGGAGTCGTTTATGAGGCCACT |
| ∆PA0575 P2 | CTCTACGGGGTACAGCTGGCGATCGAC |
| ∆PA0575 P3 | CAGCTGTACCCCGTAGAGCTCGTCGAT |
| ∆PA0575 P4 | CCTTCCTCGACGAGTGACGAA |
| ∆PA0575 P5 | CAGAGGTCGGCCGTACCC |
| ∆PA0575 P6 | ACTCCACCATGCAGTCGC |
| ΔPA0847 P1 | GGTCGATCAGGAACGAAGCAC |
| ΔPA0847 P2 | TCAGGCTGGGTGCGAATTGAAATTCGACATTTC |
| ΔPA0847 P3 | AATTCGCACCCAGCCTGAAATGCGCGG |
| ΔPA0847 P4 | GGCAGGAAGTAGGCGAGCTTGC |
| ΔPA0847 P5 | CACCATCTCGTCCACCTCAC |
| ΔPA0847 P6 | ATCATCCAGATGCCGAAGAG |
| ∆PA0861 P1 | CGCTGTCTGTTCCGCCAGGTG |
| ∆PA0861 P2 | CACGTGGCCGGCCGCCTCGATGTCCGG |
| ∆PA0861 P3 | GAGGCGGCCGGCCACGTGATGGGCAAA |
| ∆PA0861 P4 | GGCGCGCCATGTCTTCGC |
| ∆PA0861 P5 | ACCTCGGCCCTCGACGCC |
| ∆PA0861 P6 | ACTCGCCGGTGACGCCTTCGA |
| ∆PA1107 P1 | CATGCTGCGGGTCGGACT |
| ∆PA1107 P2 | TTCCGCCCGCGCGAACGCCTCGAAGGT |
| ∆PA1107 P3 | GCGTTCGCGCGGGCGGAAGTCCTTGAG |
| ∆PA1107 P4 | TGTTGGCCGCCGTCGGATA |
| ∆PA1107 P5 | CATGCTGCGGGTCGGACT |
| ∆PA1107 P6 | TGGCCGACCGCCCGCTAC |
| ∆PA1120 P1 | CGATTCGGCGCGCCGTTC |
| ∆PA1120 P2 | CGTTCCATCGCCCTCTATCCCGAGCAT |
| ∆PA1120 P3 | ATAGAGGGCGATGGAACGGGCGATCAG |
| ∆PA1120 P4 | CCGAAGGAGCCGGCGGTC |
| ∆PA1120 P5 | ATAGGCCGGAAGCGGGGT |
| ∆PA1120 P6 | CGACTACACTGGCCAGTCGTG |
| ∆PA1181 P1 | GCTGGACATCATCGAGAGCTACGA |
| ∆PA1181 P2 | GTCCAGGTTGGCCCAGAGCACGCGAAC |
| ∆PA1181 P3 | CTCTGGGCCAACCTGGACAAGAGCCCG |
| ∆PA1181 P4 | GACAGCCTGACCAGCCAG |
| ∆PA1181 P5 | CGGCAACCTGCTGGAGAACGC |
| ∆PA1181 P6 | CTCGCCAGTTGGCTGATC |
| ∆PA1433 P1 | CCGACTGTACGCTGGAGG |
| ∆PA1433 P2 | CTGGTCGAGGAAGGCGACAAGCGGTTG |
| ∆PA1433 P3 | GTCGCCTTCCTCGACCAGCGGCTTGCC |
| ∆PA1433 P4 | AACCAGGCGCACATGGTG |
| ∆PA1433 P5 | TGTTCAAGGAGCGCAAAGGCT |
| ∆PA1433 P6 | CTCGACGAAGCGGCCAAG |
| ∆PA1727 P1 | CCGCGTTGCTCATGCGAG |
| ∆PA1727 P2 | GCCCTCTGGCCGGCGAGCGAACTGAAG |
| ∆PA1727 P3 | GCTCGCCGGCCAGAGGGCGAAGGCCGA |
| ∆PA1727 P4 | AGCCAGAGGAAGATCGGACGA |
| ∆PA1727 P5 | CCACCTCGGGGTCCCAGC |
| ∆PA1727 P6 | CGAAACTGGCCCGCTGCC |
| ∆PA1851 P1 | ATACCTGCAGCGGTCCGG |
| ∆PA1851 P2 | CGCGCGGTCCCAGTCGGCCACGATGAA |
| ∆PA1851 P3 | GCCGACTGGGACCGCGCGCTCTACGAG |
| ∆PA1851 P4 | GAAAGCATCAGTTAGCGTGACCG |
| ∆PA1851 P5 | GCCGGGCACCAACAGGGT |
| ∆PA1851 P6 | GCGAAGCGGTCCGCTGCC |
| ∆PA2072 P1 | TGTACGACGGCTCCTACCAT |
| ∆PA2072 P2 | TCAAGGCCGAGCAAGCATCGATTCGCC |
| ∆PA2072 P3 | ATGCTTGCTCGGCCTTGAGCGGGCGTC |
| ∆PA2072 P4 | TGAACACGTTGAAGCCGATA |
| ∆PA2072 P5 | CTGGAGTTCCACAACGAGGT |
| ∆PA2072 P6 | GGAGACGTAGACCGCATAGC |
| ∆PA2133 P1 | AACCGGATACCGCGCTGGC |
| ∆PA2133 P2 | TCACCCCTGACCGTTCACTGTAGAGC |
| ∆PA2133 P3 | GTGAACGGTCAGGGGTGACCGGGGAGG |
| ∆PA2133 P4 | CCTGGGTGAACAGTTCCACG |
| ∆PA2133 P5 | GAGAAACTCGCGCTCGGCAG |
| ∆PA2133 P6 | AGTACCAGCAACCACAGGCTG |
| ΔPA2200 P1 | TTCGATGCGCCTGTCTATCG |
| ΔPA2200 P2 | CACGCCGATGGTGTACGAGCGCAGGTG |
| ΔPA2200 P3 | TCGTACACCATCGGCGTGGTCGTCGAA |
| ΔPA2200 P4 | CTTGAGCAGCAGCGTACTGG |
| ΔPA2200 P5 | CCGGCAAGCTGAACATCGTC |
| ΔPA2200 P6 | GGATGAGCTCCTGGGCATCG |
| ΔPA2567 P1 | ACGAGCATCGGCATGAGCAA |
| ΔPA2567 P2 | CAGGTGGAACTCGAATACCTCGTCCGC |
| ΔPA2567 P3 | GTATTCGAGTTCCACCTGCTGCAGGCA |
| ΔPA2567 P4 | GAAGGCATTCTGGCTGTTCTT |
| ΔPA2567 P5 | GTATATCTAGATGAACCGGGGGAT |
| ΔPA2567 P6 | GCCGAGTGCTGGAAACATC |
| ∆PA2572 P1 | GCCATCTGGCGGACCTCCTG |
| ∆PA2572 P2 | CTAGGTCGTGTCGACCAGCAGCACGCT |
| ∆PA2572 P3 | ATGAACGATACGACCTAGGCGGCTCCC |
| ∆PA2572 P4 | CTCGTCCGACGACTGGCCG |
| ∆PA2572 P5 | ACGTGCTCCTCCAGGGTGGT |
| ∆PA2572 P6 | GGGCGAGATGCAGGAGAACC |
| ∆PA2771 P1 | TTCCAGTTGCGCGGTGAC |
| ∆PA2771 P2 | TCAGACGCTGGCGAGCATACTGGATCC |
| ∆PA2771 P3 | ATGCTCGCCAGCGTCTGACCAGGGCTA |
| ∆PA2771 P4 | CAGGCCGATATGTTGCAGTT |
| ∆PA2771 P5 | GTTCCGGACTCCTGTATCGATG |
| ∆PA2771 P6 | ATCGCTTCGGCAAGGCTT |
| ∆PA2818 P1 | TCCCCTAGGGGACGCCATT |
| ∆PA2818 P2 | CTAGCTCGCGATCTGCATAGGTGCCAC |
| ∆PA2818 P3 | ATGCAGATCGCGAGCTAGAGGGGCAGA |
| ∆PA2818 P4 | GCCGCGAAACCCTTTATACTGC |
| ∆PA2818 P5 | GTGGCGACACCTTGGGACTCC |
| ∆PA2818 P6 | CGCCAACGCCAGCTCCAGGA |
| ∆PA2870 P1 | CTGACCCTGTCCGACCTCGAA |
| ∆PA2870 P2 | CAGGCTTTCAGTCTGCCACAGCTCGTT |
| ∆PA2870 P3 | TGGCAGACTGAAAGCCTGCTGGTGCGC |
| ∆PA2870 P4 | GCCCTATATCCGTAACGTGACCCC |
| ∆PA2870 P5 | GGCTTGGCGCGAACGGCA |
| ∆PA2870 P6 | CGTCAGCTTCGTCGGACCGCT |
| ∆PA3177 P1 | CATCCAGGTCGCGGTGCTGTTG |
| ∆PA3177 P2 | TCAGGCGCAAGGAGCCATGCGCGGAAC |
| ∆PA3177 P3 | ATGGCTCCTTGCGCCTGAGGCTTGCCG |
| ∆PA3177 P4 | CGCACGCAAGGAGGTGGTG |
| ∆PA3177 P5 | AACGCCTGGGCCTGGTGC |
| ∆PA3177 P6 | GAGAGCAACCGCAAGCCGCG |
| ∆PA3258 P1 | GACCGTCAGCCTGAACGAGGC |
| ∆PA3258 P2 | CGCAACGAATTCGCTCAGCCGTCCGCA |
| ∆PA3258 P3 | CTGAGCGAATTCGTTGCGCACAACCGC |
| ∆PA3258 P4 | CGCGAGCCTGGCAGTGGA |
| ∆PA3258 P5 | CTCCGGGCAGAGCACCCA |
| ∆PA3258 P6 | GCGCAGTTCGCTGGTCATCAG |
| ΔPA3311 P1 | CAGGAACGCCGCGAACGAGG |
| ΔPA3311 P2 | CTCGCAATGGACACGGAAAACCCGCGG |
| ΔPA3311 P3 | TTCCGTGTCCATTGCGAGCTGGTGCAG |
| ΔPA3311 P4 | AGGTCTGCAACCAGATGATCGTTG |
| ΔPA3311 P5 | CCGAGCAACAGCAGATAGCCG |
| ΔPA3311 P6 | TTGCTGGTGGACTTCTCCAGCG |
| ΔPA3343 P1 | GCTGCGCCGCGACCTCAAACC |
| ΔPA3343 P2 | TGGCCCAGGTCAGCGGGGTCG |
| ΔPA3343 P3 | TCAGGCCACCACGCACACCTCTTCTCT |
| ΔPA3343 P4 | GTGTGCGTGGTGGCCTGAGTCCATGGC |
| ΔPA3343 P5 | CCTGGCGCAGTTCGCAGACG |
| ΔPA3343 P6 | CGTGATGGGCGAAGGCGTCC |
| ΔPA3702 P1 | GTGGTGCTGATGGACCTGCT |
| ΔPA3702 P2 | CGTGGAACAGCATTCAATTT |
| ΔPA3702 P3 | AAAGATACCCCCGAATGGTC |
| ΔPA3702 P4 | GCGGTGTCCTTGTTGGCGT |
| ΔPA3702 P5 | GAGGATCGGAATCGTCAATG |
| ΔPA3702 P6 | CTGCATTTCCAGCTCGTACA |
| ∆PA3825 P1 | AGGATGCCTTGCTGGTAGA |
| ∆PA3825 P2 | GCCTCGGTCTGATCGCCACTTGCTCGC |
| ∆PA3825 P3 | TGGCGATCAGACCGAGGCTAGCGGCTA |
| ∆PA3825 P4 | GAAAGGCCGCGTAGCTTAC |
| ∆PA3825 P5 | CCAGGAGGCGATGGCGATC |
| ∆PA3825 P6 | CAGGACTTCCAGCTTGCCGC |
| ΔPA3947 P1 | GGTCTTCTTCCTTGTTGAC |
| ΔPA3947 P2 | GTGCTGTTCCTCATCCTCCAACACCAG |
| ΔPA3947 P3 | GAGGATGAGGAACAGCACTTTCTCGAC |
| ΔPA3947 P4 | TTTTCCCCCATCCGCTTGA |
| ΔPA3947 P5 | GGACGATCATCTCGCGGTCG |
| ΔPA3947 P6 | GCGCTGAGCACATCGTCG |
| ΔPA4108 P1 | GCCGCTACCACACTTGCCTT |
| ΔPA4108 P2 | ATAAGAGCCGAGGCGAAGCTGGGTTAC |
| ΔPA4108 P3 | CTTCGCCTCGGCTCTTATTTCGACGCGGG |
| ΔPA4108 P4 | AGGCGATTCAAGTGTCGATGAAG |
| ∆PA4108 P5 | CAGTGCTGGCCTTGCTCCAG |
| ∆PA4108 P6 | CTCGCTGGCCATGCTCGAGG |
| ΔPA4332 P1 | TATCGTCGACGCTCATGGTA |
| ΔPA4332 P2 | TTCCAGCTGAGCGGACCGCGGATTTATC |
| ΔPA4332 P3 | CGGTCCGCTCAGCTGGAAAGCTGTTGCGA |
| ΔPA4332 P4 | TCAGGATCGAAAGGCTGCAA |
| ΔPA4332 P5 | ATGGTCGGTACGGTTTTCAG |
| ΔPA4332 P6 | CTCCACAGCAGCAGGTGCT |
| ΔPA4367 P1 | GCTGGCTGGTGAAGAAGG |
| ΔPA4367 P2 | GCTGTACAGCAGTTTCAAGGGGCCTTCCT |
| ΔPA4367 P3 | TTGAAACTGCTGTACAGCAAGCCGCTGC |
| ΔPA4367 P4 | GTCGCCAGGGACTCGATG |
| ΔPA4367 P5 | GGCTCCTTCGACAAGTTCAA |
| ΔPA4367 P6 | CTCTACCGCTACACCGAGGA |
| ∆PA4396 P1 | GCATGACTAGTGTCGAGCATCTGCTCGAGCATG |
| ∆PA4396 P2 | CAGGTTGAGCTGGTAGCCGGCCTGGCT |
| ∆PA4396 P3 | GGCTACCAGCTCAACCTGAAGGCCTTC |
| ∆PA4396 P4 | GCATGGGATCCCAGTTCGGCGTTGCCGGC |
| ∆PA4396 P5 | GTGACGCGCACCTGCTCG |
| ∆PA4396 P6 | CAGCTGCGTCAGCCAGGC |
| ∆PA4601 P1 | AGTCCTCACCGGCCCTGCGAC |
| ∆PA4601 P2 | GGAGTAGCCGGGCAGCGTCGTTTCCGG |
| ∆PA4601 P3 | ACGCTGCCCGGCTACTCCTCGCTGAAC |
| ∆PA4601 P4 | AGGCGATCAGCTCGATGTGCT |
| ∆PA4601 P5 | ACGGCGGCCGTGTTCACCGAA |
| ∆PA4601 P6 | GGCTCATGCCGAGGATGGTGT |
| ∆PA4781 P1 | TGGCGGTGTTGCTCGGCA |
| ∆PA4781 P2 | CTAGGCCGGGCTCTCCATCGCCCCTCC |
| ∆PA4781 P3 | ATGGAGAGCCCGGCCTAGTACGCCGAT |
| ∆PA4781 P4 | CAACACGCCATCGAGGAAAT |
| ∆PA4781 P5 | GGGCCTTCGGCTCGGTGTGG |
| ∆PA4781 P6 | CATGTTGCCGACGTCGCGTT |
| ∆PA4843 P1 | GGTACGCACATCATCCACGG |
| ∆PA4843 P2 | CACCCGGTCGATCACTCGCTGGGCGAA |
| ∆PA4843 P3 | CGAGTGATCGACCGGGTGATCAAGGGC |
| ∆PA4843 P4 | TCGAGCAACTGGAAGGGCCG |
| ∆PA4843 P5 | CCCAGCTGGGCATGTTGTCC |
| ∆PA4843 P6 | CCATTGTTCCAGGTTGCCGGTG |
| ΔPA4929 P1 | CCGAGCTTCATGAAGTCGTCCTTC |
| ΔPA4929 P2 | ATAGCCGGCAAGAGGATGGCGCACGAT |
| ΔPA4929 P3 | CATCCTCTTGCCGGCTATGCGATGGCT |
| ΔPA4929 P4 | GCCGACCTCAGTGCTGCCGA |
| ΔPA4929 P5 | TAATGGGCGATGCGCCGCAA |
| ΔPA4929 P6 | CCGGTGTGCGCGAGCTGAA |
| ∆PA4959 P1 | GCCGGGTGCAGATCAACC |
| ∆PA4959 P2 | ATCGATCTTGGCCACCAGCTGGATGAA |
| ∆PA4959 P3 | CTGGTGGCCCTGAAGCACCTGACCGTG |
| ∆PA4959 P4 | CCTGGAGCAGACCATCGC |
| ∆PA4959 P5 | ATTGCCTGAGCTGGCCGA |
| ∆PA4959 P6 | TCTGACCGAGGGTGCGGA |
| ∆PA5017 P1 | CATAATGCACCCTTTGCCTGC |
| ∆PA5017 P2 | CATGGCGATGTCCAGGGACGCCTTCAG |
| ∆PA5017 P3 | TCCCTGGACATCGCCATGGCCCACAAC |
| ∆PA5017 P4 | CGTGTAGCCGCCGGCATA |
| ∆PA5017 P5 | AACTGCGTCCCGTCGTAC |
| ∆PA5017 P6 | GAAGCTCTCCCGGCTGGC |
| ∆PA5295 P1 | GGGGATTGAGTCGGGACATAGGA |
| ∆PA5295 P2 | GTGCGCCAGAAGCTGTTCGGCAGCCAG |
| ∆PA5295 P3 | GCCGAACAGCTGGCGCACAACCTCAAC |
| ∆PA5295 P4 | CGTCTCGCTGCTGGCGAT |
| ∆PA5295 P5 | GTCCCCGGAATACTCCTTCTGC |
| ∆PA5295 P6 | GCCGGCCTATGCCGAGTAC |
| ΔPA5442 P1 | TTCTCGCTCAGCCCGGCC |
| ΔPA5442 P2 | CAACTGGCGGACGGTCATAGGCGCTTG |
| ΔPA5442 P3 | ATGACCGTCCGCCAGTTGCGCGAGATG |
| ΔPA5442 P4 | CGGCAGGGTATCGCAGACAC |
| ΔPA5442 P5 | AGTCGATCACCCCGGCGC |
| ΔPA5442 P6 | CCAGTTCGGCGAGCGGCGA |
| ∆PA5487 P1 | CTCTCGGCGCTCGCACCC |
| ∆PA5487 P2 | GCCGCCGTCCATGCACTGGTCGACCGCC |
| ∆PA5487 P3 | CAGTGCATGGACGGCGGCGATCTGCTG |
| ∆PA5487 P4 | CCAGAGCGGGACAGCCGGTC |
| ∆PA5487 P5 | CAGCCTGCGTTCCGGCTAC |
| ∆PA5487 P6 | GAGGCCGCTGAGGATGTGCT |
